# SPRED3 regulates the NF-κB signaling pathway in thyroid cancer and promotes the proliferation

**DOI:** 10.1038/s41598-024-61075-6

**Published:** 2024-09-03

**Authors:** Zhiping Chen, Congren Wang, Mingzhu Li, Shaoyang Cai, Xiaoyu Liu

**Affiliations:** Department of Thyroid Surgery, Quanzhou First Hospital Affiliated to Fujian Medical University, Quanzhou, 362000 Fujian China

**Keywords:** SPRED3, Thyroid cancer, NF-κB, Proliferation, Prognosis, Cancer epigenetics, Growth factor signalling

## Abstract

*SPRED3* (Sprouty-related EVH1 domain containing 3) mutants are depicted in various cancers, however, nothing is known about its biofunction in thyroid cancer (THCA). Bioinformatic analyses were conducted to ascertain the level of *SPRED3* expression in THCA tissues and its importance in the prognosis of THCA patients. Flag-*SPRED3* plasmid and *SPRED3*-knockout vector were developed to overexpress or deplete the SPRED3 expression in THCA cells. The function of SPRED3 on THCA cell proliferation was examined using the colony formation assay and CCK8 assay. The effect of SPRED3 expression on the transcriptional activity of NF-κB was also examined using luciferase reporter assays. High *SPRED3* expression was associated with unfavorable clinical outcomes, advanced tumor characteristics, and traditional molecular markers of papillary thyroid cancer in THCA patients. Genetic analysis revealed differences in mutation rates in key genes between SPRED3-high and SPRED3-low THCA cases. It is also revealed that SPRED3 influenced the immune microenvironment, with increased stromal and immune scores and altered immune cell infiltration. Functionally, SPRED3 overexpression enhanced THCA cell viability and colony formation, while its depletion reduced cell growth and proliferation. In vivo experiments in mice confirmed the inhibitory effect of SPRED3 depletion on tumor growth. Mechanically, we found that SPRED3 activated the NF-κB signaling. For the first time, we found that SPRED3 promotes THCA cell proliferation via the NF-κB signaling pathway. This finding may provide insight into SPRED3’s prognostic potential in thyroid cancer and provide the rationale for SPRED3-targeted druggable interventions.

## Introduction

Thyroid cancer (THCA) represents a common malignancy within the endocrine system. Over the past few decades, the incidence of THCA has increased more than fourfold^1^. Currently, THCA affects 586,000 individuals globally, ranking it as the ninth most prevalent form of cancer^2^. Conventional surgical thyroidectomy and relative targeted therapies have yielded favorable outcomes, with a 99% 5-year survival rate for THCA patients^3^. Nonetheless, these treatments have exhibited limited clinical benefits for aggressive THCA cases^4^. Thus, addressing these challenges necessitates a comprehensive understanding of the underlying mechanisms involved in THCA pathogenesis.

The Sprouty-related EVH1 domain containing 3 (*SPRED3*), also known as Eve-3 and spred-3, is characterized by the presence of a C-terminal Sprouty-like cysteine-rich domain (SRY) and an N-terminal Ena/Vasodilator-stimulated phosphoprotein (VASP) homology-1 (EVH-1) domain. Belonging to the Sprouty-related protein family, SPRED3 negatively regulates the MAPK (mitogen-activated protein kinase) pathway^5^. Notably, the SPRED3 protein facilitates protein kinase binding, potentially impeding the activation of the Ras/MAPK cascade^6^. Studies have identified multiple SPRED3 mutations in cervical carcinoma and glioblastoma^7^. Moreover, aberrant methylation of SPRED3 has prognostic implications for neuroblastoma patients^8^. Furthermore, bioinformatics analysis has revealed SPRED3 to be a downstream transcription factor of the WNT signaling pathway, which is well-documented for its oncogenic role in various cancers^9^. However, the specific role of SPRED3 in THCA remains elusive.

The nuclear factor-κB (NF-κB) transcription factor governs cell fate by regulating the transcription of numerous genes^10,11^. Sustained constitutive activation of NF-κB promotes oncogenesis, tumor progression, and metastasis^12,13^. Abnormally high NF-κB activity is ubiquitously found in different cancers, acting as clinical hallmarks of chronic inflammation and tumorigenesis^14,15^. Targeting NF-κB therapeutically has emerged as a promising strategy against various cancers, as inhibiting NF-κB signaling has been shown to induce tumor cell death^16–18^.

In the current work, we used several bioinformatics analyses to explore SPRED3 expression levels in THCA patients for the first time, and we subsequently evaluated the impact of *SPRED3* overexpression or depletion on THCA cell proliferative behaviors. Finally, the mechanism for the SPRED3-mediated THCA progression was also investigated, which will shed light on a novel therapy for THCA and facilitate the exploration of the THCA biomarkers or therapeutic targets.

## Results

### SPRED3 upregulation in THCA tissues predicts an unfavorable clinical outcome

**Figure 1:**
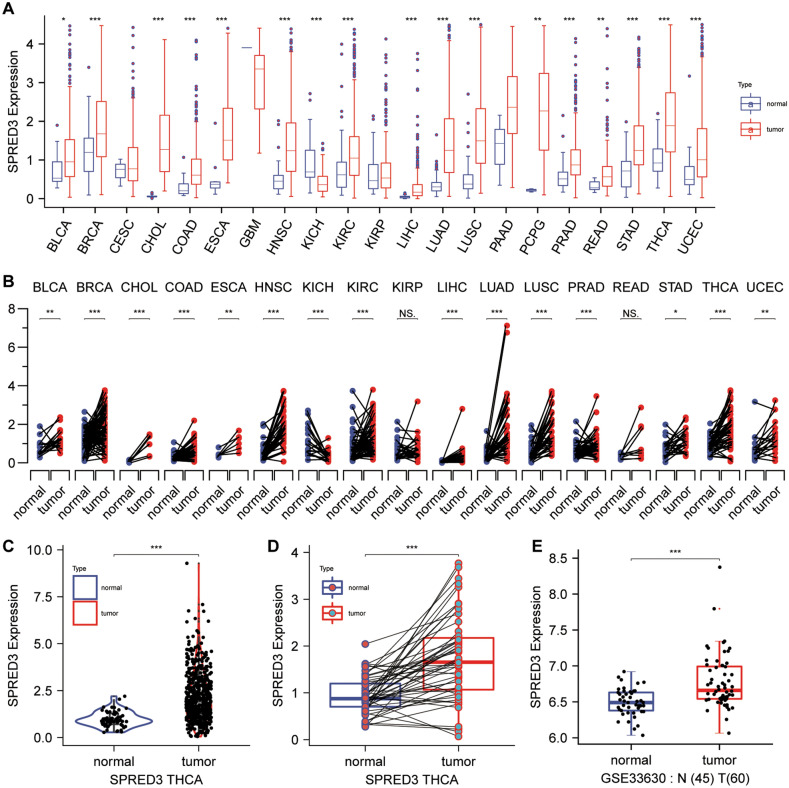
*SPRED3* mRNA level is enhanced in THCA. (**A**) *SPRED3* mRNA expression was analyzed in TCGA-pan-cancer tissues and TCGA normal tissues. (**B**) *SPRED3* mRNA expression was analyzed in THCA-pan-cancer tissues and corresponding normal tissues. (**C**) *SPRED3* mRNA expression was analyzed in TCGA-THCA tissues and TCGA normal tissues. (**D**) *SPRED3* mRNA expression was analyzed in TCGA-THCA tissues and TCGA normal tissues. (**E**) *SPRED3* mRNA expression in THCA tissues and normal tissues was analyzed based on GSE33630.

**Figure 2:**
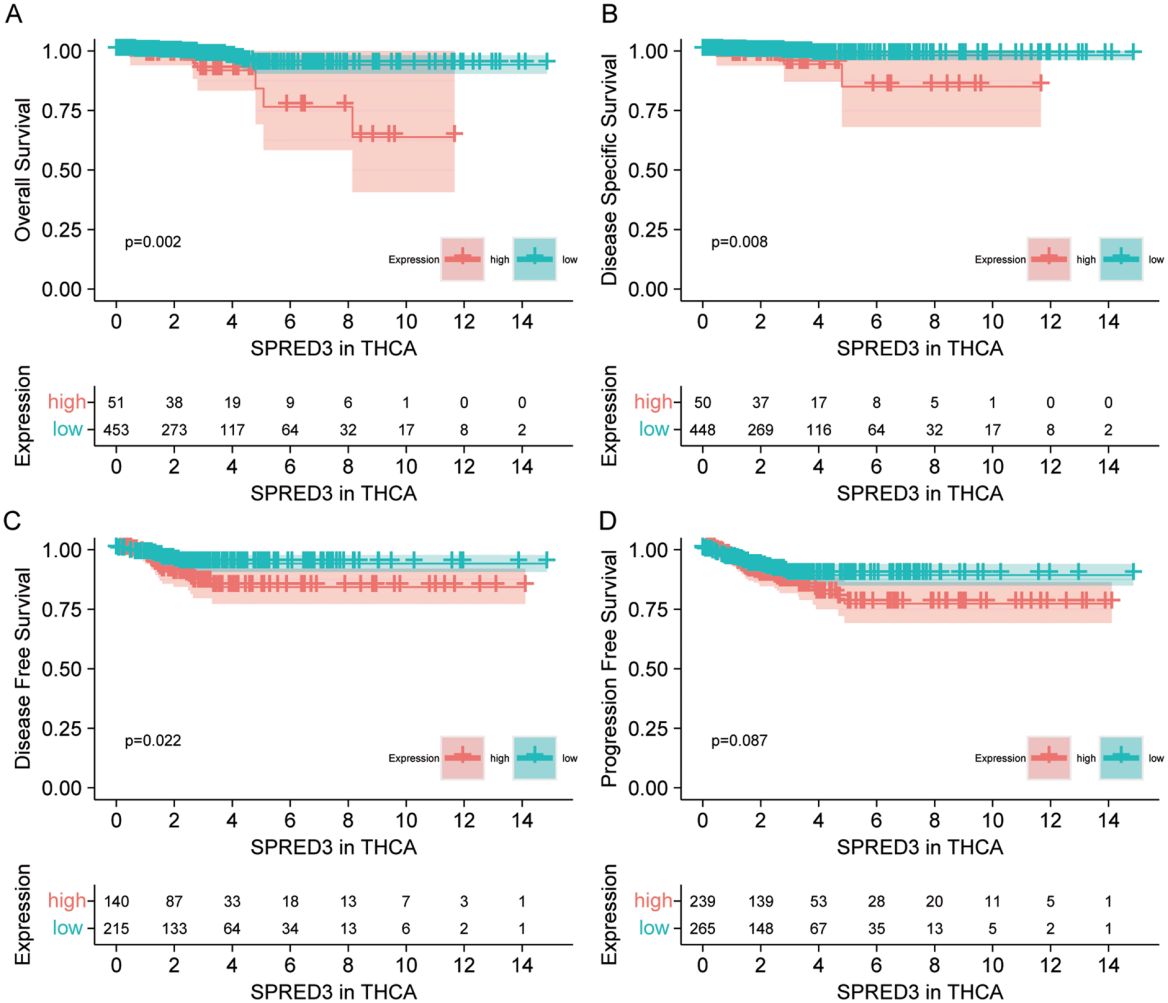
The prognostic value of *SPRED3* expression in TCGA-derived THCA patients. The patients were grouped into *SPRED3*-high and *SPRED3*-low patients. (**A**) Overall survival. (**B**) Disease-specific survival. (**C**) Disease-free survival. (**D**) progression-free survival.

**Figure 3:**
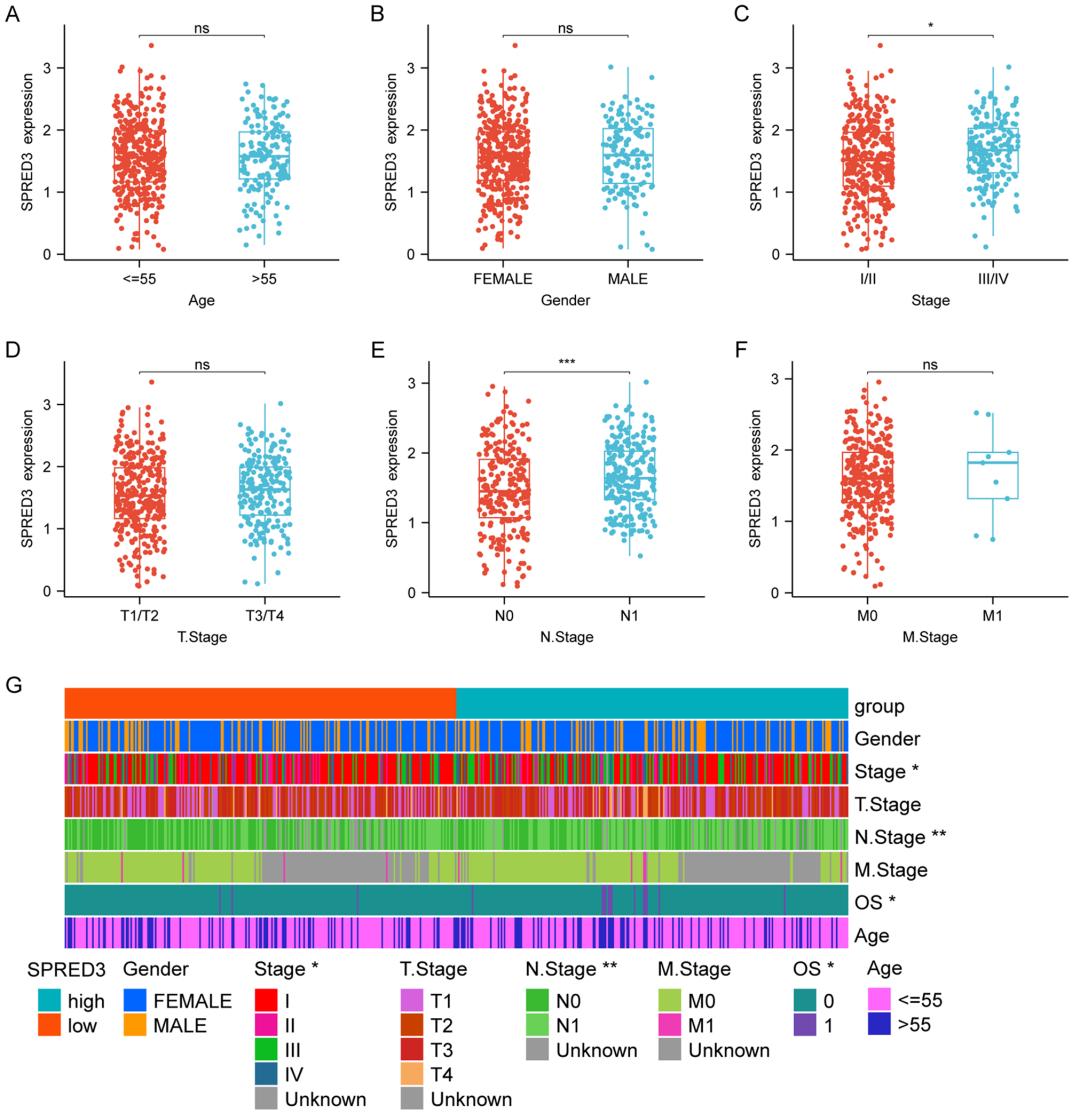
Correlation with clinicopathological characteristics of THCA patients with *SPRED3* expression.

**Figure 4:**
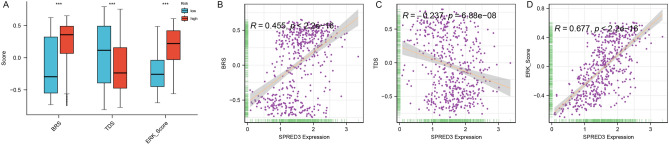
Relationship of *SPRED3* expression with BRS, TDS and ERK score. (**A**) *SPRED3* expression between high and low BRS, TDS and ERK tumor groups. (**B**) Pearson correlation analysis determining the correlation of *SPRED3* expression with BRS level. (**C**) Pearson correlation analysis determining the correlation of *SPRED3* expression with TDS level. (**D**) Pearson correlation analysis determining the correlation of *SPRED3* expression with ERK level.

The expression of SPRED3 was assessed in TCGA pan-cancer tissues and normal paratumor tissues. The results indicated an overall increase in *SPRED3* expression across most cancer types (Fig. 1A,B). Particularly, THCA tissues exhibited robust SPRED3 expression compared to corresponding normal tissues and tumor-free tissues from the TCGA database (Fig. 1C,D). Consistently, GSE33630 also demonstrated elevated SPRED3 mRNA levels in THCA tissues (Fig. 1E). Survival analysis revealed a significant association between high *SPRED3* levels and unfavorable clinical outcomes in THCA patients (Fig. 2). Notably, high *SPRED3* expression correlated with pathological prognostic indicators of THCA, including advanced tumor depth (T stage) and nodal involvement (N-Stage) (Fig. 3 and Table 1). In addition, we estimated the *SPRED3* expression difference in the traditional molecular markers of papillary thyroid cancer such as BRAFV600E-RAS score (BRS), Thyroid differentiation score (TDS) and ERK activation level (i.e., ERK score). According to the TDS, BRS, ERK scores, we divided the papillary THCA patients into low and high TDS, BRS or ERK tumor groups. Comparison of *SPRED3* expression in the high and low groups showed that *SPRED3* expression was higher in the high BRS or ERK tumor groups, while lower in the high TDS tumor groups (Fig. 4A). These observations were corroborated by the findings of the TGCA network, showing the *SPRED3* expression was positively with BRS or ERK score, while negatively with TDS score (Fig. 4B–D).

**Table 1:** Correlation with clinicopathological characteristics of THCA patients with SPRED3 expression.

	Level	High	Low	p
n		252	252	
Age (%)	≤ 65	214 (84.92)	219 (86.90)	0.6085
	> 65	38 (15.08)	33 (13.10)	
Gender (%)	Female	184 (73.02)	185 (73.41)	1
	Male	68 (26.98)	67 (26.59)	
Stage (%)	Stage I	134 (53.39)	149 (59.36)	0.0395
	Stage II	20 (7.97)	32 (12.75)	
	Stage III	67 (26.69)	45 (17.93)	
	Stage IV	30 (11.95)	25 (9.96)	
M (%)	M0	147 (96.71)	135 (97.12)	1
	M1	5 (3.29)	4 (2.88)	
N (%)	N0	100 (43.48)	129 (57.59)	0.0036
	N1	130 (56.52)	95 (42.41)	
T (%)	T1	67 (26.69)	76 (30.28)	0.476
	T2	79 (31.47)	87 (34.66)	
	T3	92 (36.65)	78 (31.08)	
	T4	13 (5.18)	10 (3.98)	
Type (%)	High	252 (100.00)	0 (0.00)	< 0.0001
	Low	0 (0.00)	252 (100.00)

**Figure 5:**
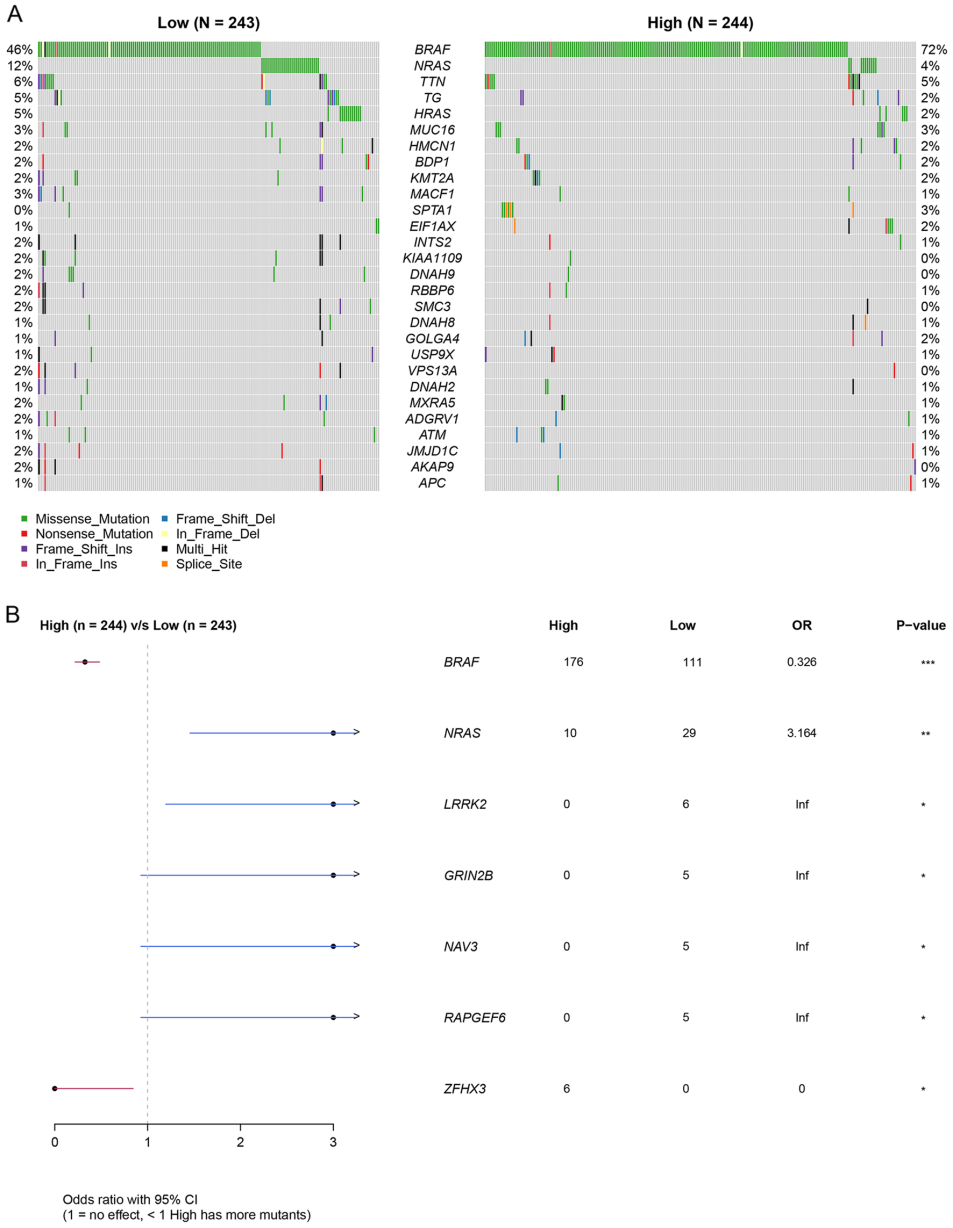
Oncoplots (**A**) and forest plots (**B**) demonstrating the top genetic mutation in *SPRED3*-low and *SPRED3*-high THCA patients.

To gain insights into the mutational landscape, TCGA DNA sequencing data was utilized to compare genetic mutations between *SPRED3*-low and *SPRED3*-high THCA patients. The analysis revealed higher mutation rate in the BRAF in *SPRED3*-high THCA patients versus in the SPRED3-low THCA patients (72% versus 46%) and the lower mutation rate in the BRAF in *SPRED3*-high THCA patients versus in the SPRED3-low THCA patients (5% versus 12%) (Fig. 5A). Further univariate and multivariate analysis showed that SPRED3-high THCA patients had more mutants in BRAF (B-Raf proto-oncogene, serine/threonine kinase, p < 0.0001) and ZFHX3 (zinc finger homeobox 3, p < 0.05) as well as less mutants in LRRK2 (leucine rich repeat kinase 2, p < 0.05), GRIN2B (glutamate ionotropic receptor NMDA type subunit 2B, p < 0.05), NAV3 (neuron navigator 3, p < 0.05), and RAPGEF6 (Rap guanine nucleotide exchange factor 6, p < 0.05) (Fig. 5B).

**Figure 6:**
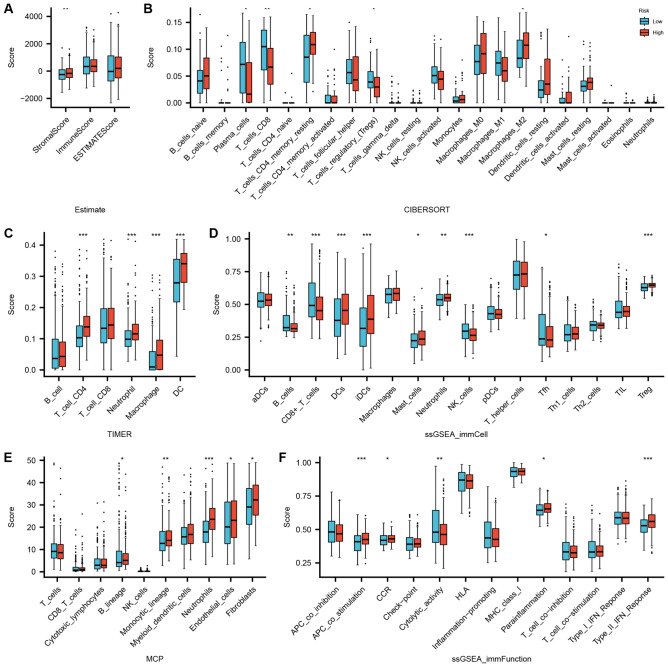
Analysis of immune microenvironment with *SPRED3* expression using the ESTIMATE algorithm (**A**) and CIBERSORT (**B**), TIMER (**C**), ssGSEA_immCell (**D**), MCPcounter (**E**), and ssGSEA_immuFunction (**F**).

Given the reported role of SPRED family proteins as crucial modulators of immunity, the TCGA THCA cohort was stratified into two groups based on median *SPRED3* expression in THCA. Univariate analysis was employed to assess the differences in the immune microenvironment and immune cell infiltration between the groups. Significantly distinct immune landscapes were observed (Fig. 6). The SPRED-high group exhibited higher stromal scores, immune scores, and estimate scores compared to the SPRED-low group. Furthermore, analysis using the TIMER database revealed elevated scores of B lineage, monocytic lineage, and neutrophils in the *SPRED3*-high group. Collectively, these findings underscore the involvement of *SPRED3* in THCA malignancy.

### Overexpressing SPRED3 drives the cell viability of THCA cells

**Figure 7:**
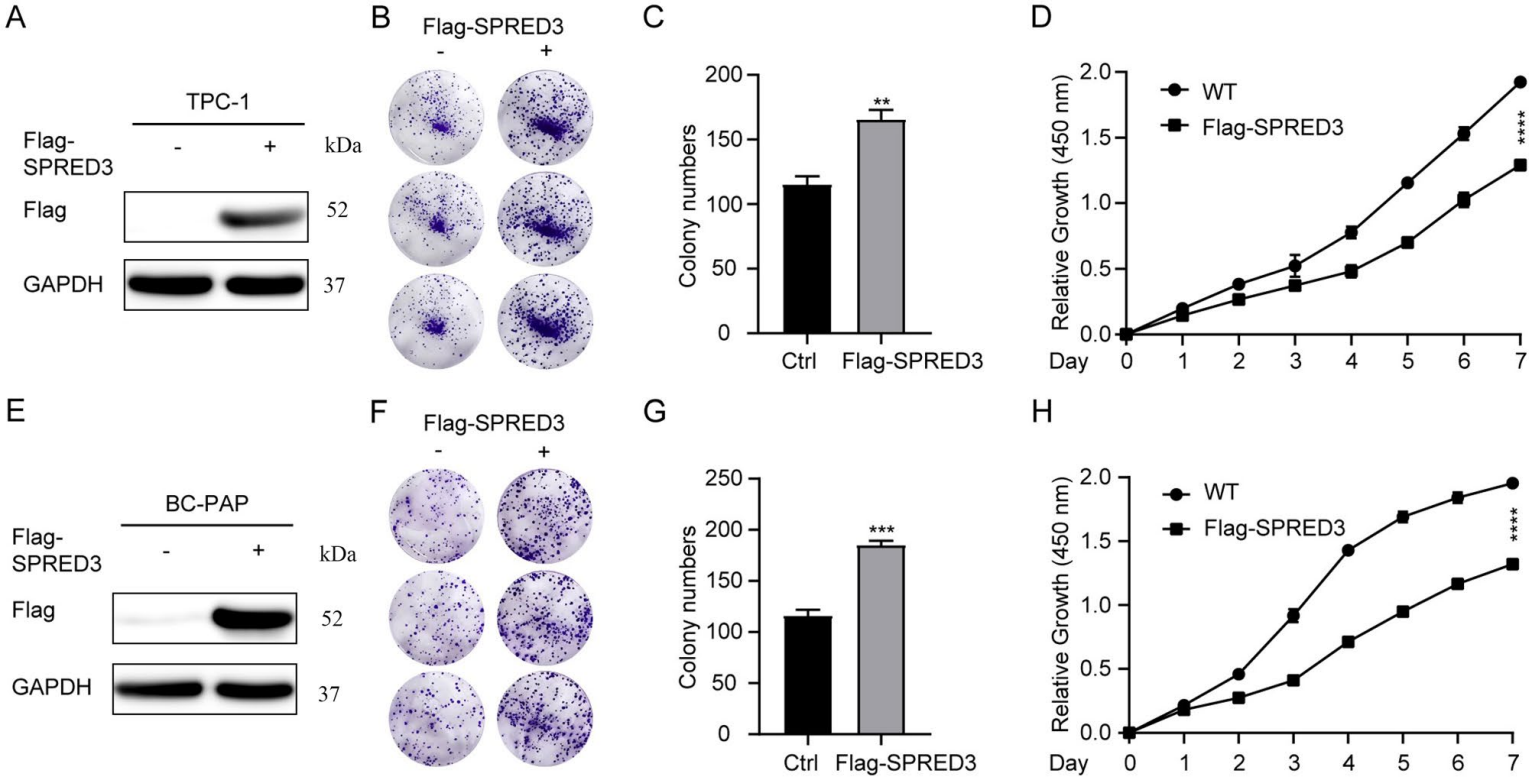
*SPRED3* overexpression promotes cell proliferation in THCA cells. (**A**) SPRED3 overexpression was verified by western blot analysis in TPC-1 cells. (**B**,**C**) The proliferation was assessed by colony formation assay. (**D**) Cell proliferative rate was assessed by CCK8 assays. (**E**) SPRED3 overexpression was verified by western blot analysis in BC-PAP cells. (**F**,**G**) The viability of SPRED3-overexpressing and normal BC-PAP cells was examined by colony formation assay. (**H**) Cell proliferative rate of SPRED3-overexpressing and normal BC-PAP cells was assessed by CCK8 assays.

We overexpressed the SPRED3 in TPC-1 cells as verified by western blotting (Fig. 7A). Utilizing the CCK8 and colony formation tests, we subsequently assessed SPRED3’s impact on the viability of THCA cells. As seen in Fig. 7B–D, overexpressing SPRED3 enhanced viability and colony formation rate. CCK8 and colony formation assays confirmed that SPRED3 overexpression also increased BCP-AP proliferation (Fig. 7E–H).

### Depleting SPRED3 curbs THCA growth

**Figure 8:**
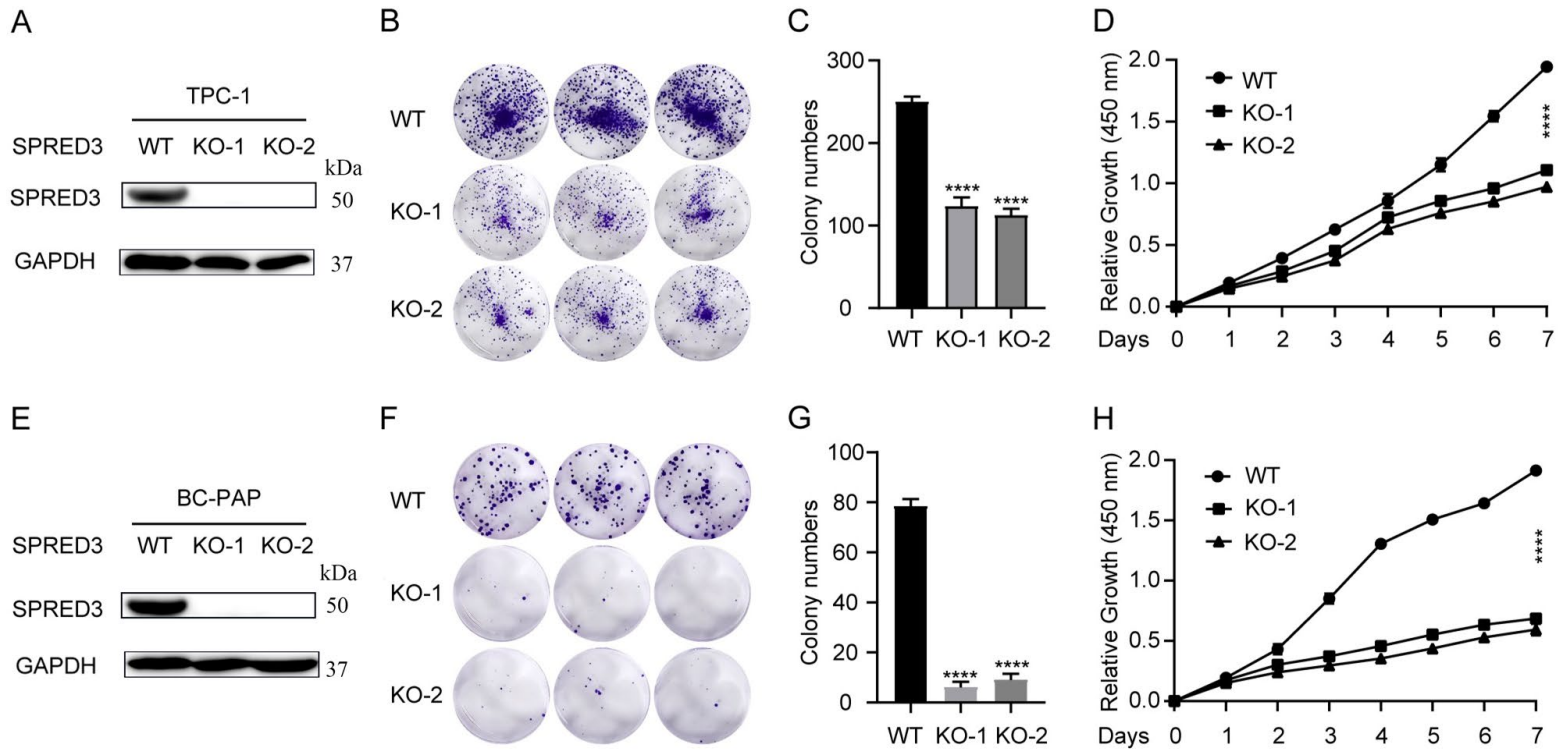
*SPRED3* deficiency lessens cell proliferation in THCA cells. (**A**) SPRED3 deficiency was verified by western blot analysis in TPC-1 cells. (**B**,**C**) Colony formation assay assessing TPC-1 cell proliferation when SPRED3 deficiency or not. (**D**) Cell proliferative rate of SPRED3-deficient and normal TPC-1 cells was assessed. (**E**) SPRED3 deficiency was verified by western blot analysis in BC-PAP cells. (**F**,**G**) The viability of SPRED3-deficient and normal BC-PAP cells were examined by colony formation assay. (**H**) Cell proliferative rate of SPRED3-overexpressing and normal BC-PAP cells was assessed by CCK8 assays.

**Figure 9:**
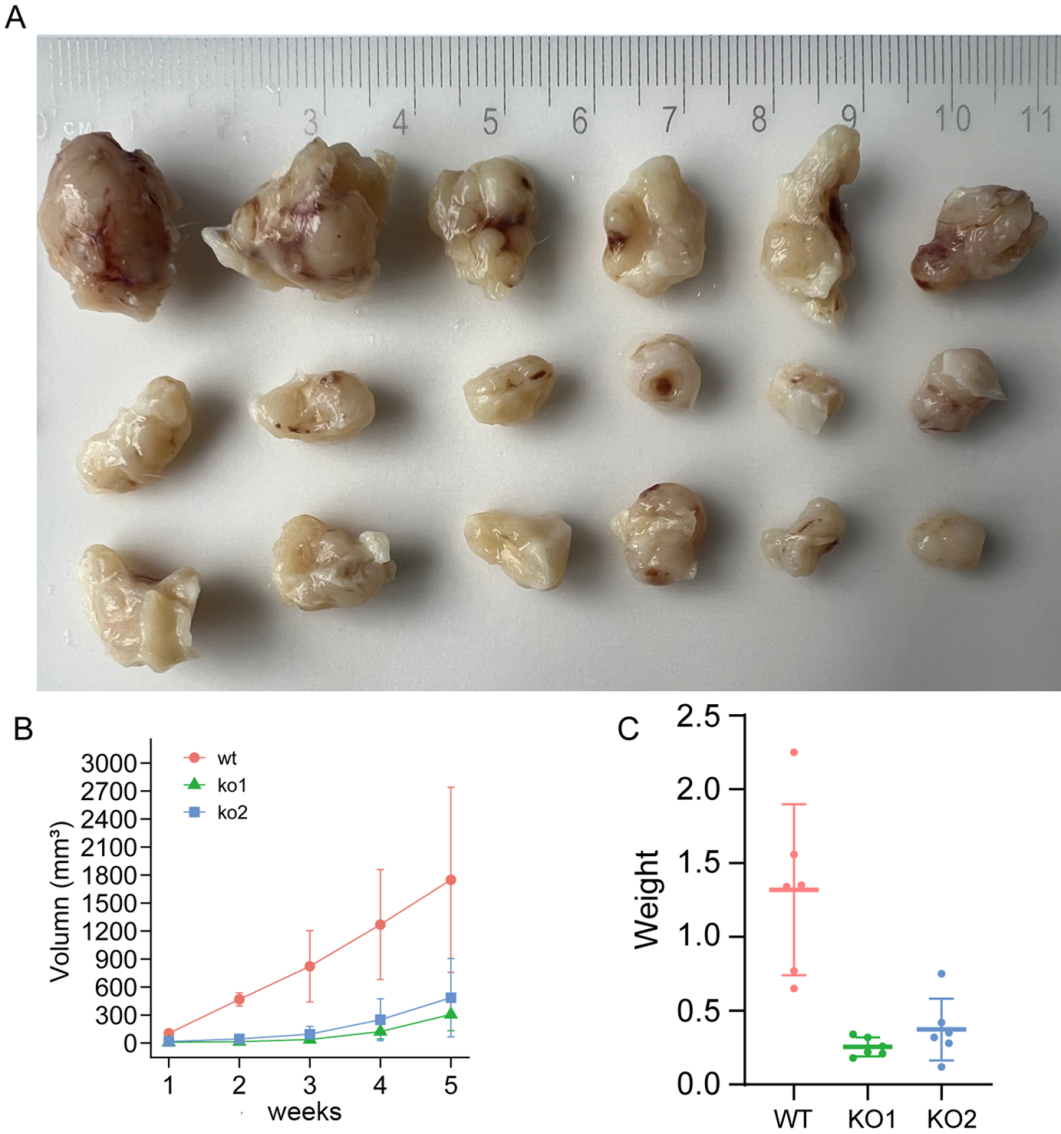
SPRED3 deficiency lessens THCA tumor growth in vivo. SPRED3-deficient THCA cell clones were subcutaneously implanted into two KO1 and KO2 groups. Wild-type THCA cells were implanted into the WT group of mice. The tumor size was tested every week. The tumor was detached and weighed after the mice had received the prescribed amount of CO_2_ asphyxiation. (**A**) Representative pictures of the Xenograft tumor. (**B**) Plotting Xenograft tumor volumes. (**C**) Plotting Xenograft tumor weight.

We created SPRED3-deficient TPC-1 cells by utilizing the CRISPR/Cas9-mediated knockout technique (Fig. 8A). Figure 8B–D demonstrates that as compared to normal cells, two SPRED3-deficient TPC-1 cell clones (KO-1 and KO-2) had lower rates of colony formation and cell proliferation. Comparing SPRED3-deficient BC-PAP cells to untransfected cells revealed the same declining tendency in the proliferation rate (Fig. 8E–H). We then explored the SPRED3 depletion on the THCA tumorigenesis in vivo by subcutaneous injection of KO-1 and KO-2 into the mice. The tumor weights were measured in 1,2,3,4, and 5 weeks and tumor sizes was recorded after all animals were euthanized. The results showed that SPRED3 depletion retarded tumor growth (Fig. 9A,B) and reduced the tumor weight compared with the WT group (Fig. 9C).

**Figure 10:**
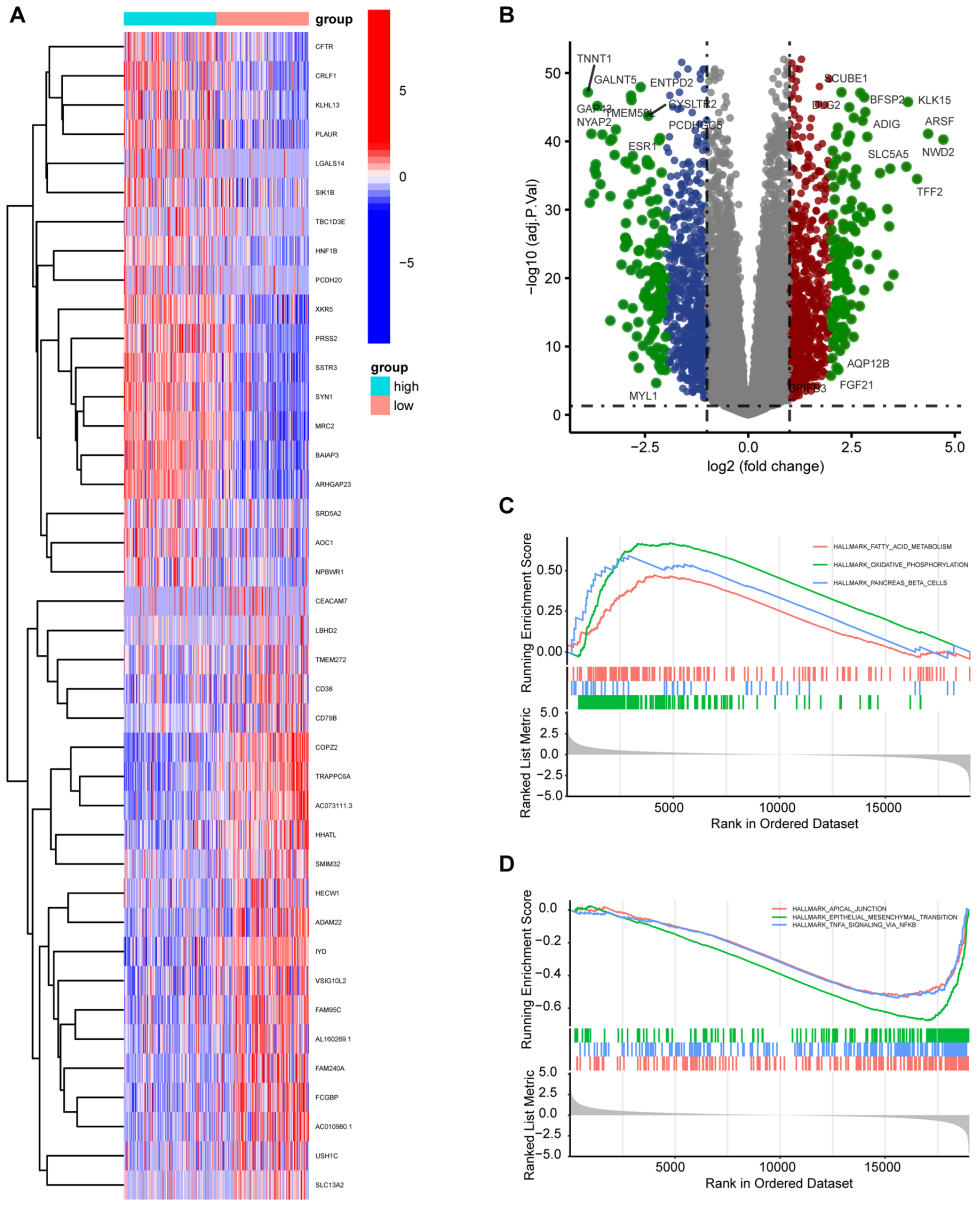
GESA analyzes the enrichment of associated signaling pathways. (**A**) Heatmap showing differential expression patterns of the genes between THCA patients with high and low *SPRED3* expression. (**B**) Volcano plot demonstrating differential expression between THCA patients with high and low SPRED3 expression. (**C**) GSEA identifies the positive signaling pathways involved in highly expressed *SPRED3*. (**D**) Based on the pathways presented in the curated gene set enrichment analysis, GSEA was carried out to find significantly enriched pathways that were different between the high (top 10%) and low (bottom 10%) *SPRED3*-expressing groups.

**Figure 11:**
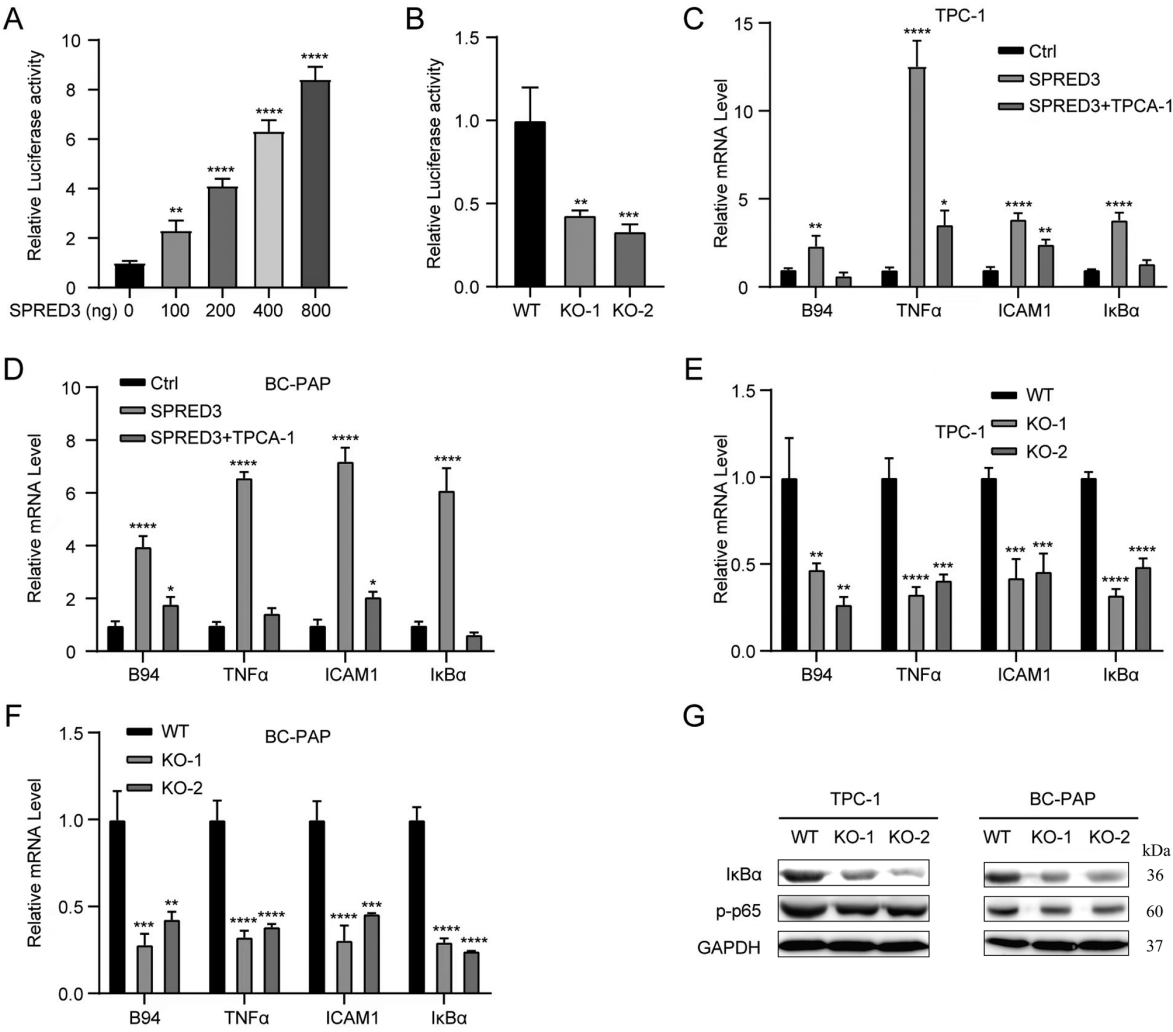
*SPRED3* overexpression activates the NF-κB signaling pathway. (**A**) Dual-luciferase reporter gene assays determining the transcriptional activity of NF-κB when ectopic expression of *SPRED3* in 293T cells. (**B**) transcriptional activity of NF-κB in SPRED3-deficient THCA cells. (**C**,**D**) *TNFα*, *B94*, *ICAM1, Iκba* mRNA in THCA cell when SPRED3 targeted depletion or overexpression by RT-qPCR when TPCA-1 copresented. (**E**,**F**) *TNFα*, *B94*, *ICAM1, Iκba* mRNA in THCA cell when SPRED3 targeted depletion by RT-qPCR. (**G**) Iκba expression was determined by Western blot.

### SPRED3 triggers NF-κB signaling cascades

Volcano plots and heatmap plots of differentially expressed mRNAs are shown in Fig. 10A,B. To elucidate the underlying mechanism behind the *SPRED3* oncogenic function, we analyzed the signaling pathway difference between the high (top 10%) and low (bottom 10%) *SPRED3*-expressing groups based on pathways based on the RNA sequence data from TCGA. SPRED3 had a positive enrichment for fatty acid metabolism, oxidative phosphorylation, pancreas beta cells and a negative enrichment for genes associated with apical junction, epithelial–mesenchymal transition, and TNFα signaling via NF-κB (Fig. 10C,D). Therefore, we further determined how *SPRED3* regulated the NF-κB signaling pathway since the involvement of NF-κB in cellular malignant behaviors^19^. To address it, we co-transfected *SPRED3* overexpression plasmids and NF-κB-Luc reporter plasmids into 239T cells. As shown in Fig. 11A, *SPRED3* induced the transcriptional activation of NF-κB in a dose-dependent manner. The diminished transcriptional activities of NF-κB were also observed in two *SPRED3*-deficient 293T cells (Fig. 11B). The four important NF-κB downstream effectors *B94* (TNFAIP2 (B94) TNF alpha induced protein 2), *TNFα* (tumor necrosis factor alpha), *ICAM1* (intercellular adhesion molecule 1), and *IκBα* (NF-κB inhibitor alpha) were next evaluated in *SPRED3*-overexpressing TCHA cell clones and wild-type THCA cells after exposure to TPCA-1 (one NF-B inhibitor)^20^. *SPRED3*-enforced expression increased the expression of the four critical downstream effectors of the NF-κB signaling pathway, *B94*, *TNFα*, *ICAM1*, and *IκBα*, this promoting effect was abrogated by TPCA-1 (Fig. 11C,D). Unsurprisingly, targeted depletion of *SPRED3* reduced *B94*, *TNFα*, *ICAM1*, and *IκBα* expression (Fig. 11E,F). Western blot analysis manifested that *SPRED3* deficiency in KO THCA cells led to the reduction of *IκBα* expression (Fig. 11G).

## Discussion

In this study, we observed a substantial elevation of *SPRED3* expression in THCA tissues and emphasized its potential as an independent prognostic indicator for THCA patients. Furthermore, we found the tight relationship between *SPRED1* and the immune microenvironment, as well as the differentiation and function of immune cells. Notably, highly-expressed *SPRED1* was correlated with increased rates of genomic mutation. Of significant importance, our data revealed that *SPRED3* facilitated THCA growth through activation of the NF-κB signaling pathway, suggesting a promising therapeutic target for THCA intervention.

Sprouty (Spry)/Spred protein is recognized as “downregulating” MAPK signaling^21,22^, which is highly oncogenic in cancers^23,24^. Their mutants are detected in patients with neurofibromatosis and cervical carcinoma^25,26^. Disabled SPRED3 confers resistance to the epidermal growth factor receptor by stimulating the MAPK cascades in non-small-cell lung cancer^6^. Therefore, SPRED3 might be a tumor suppressor during cancer malignancy. Herein, we found that *SPRED3* was markedly elevated in TCGA-THCA specimens and was connected to poor clinical consequences. Furthermore, high-*SPRED3* THCA patients had higher immune cell infiltration which is important for survival and tumor metastasis in patients. The high *SPRED3* expression was also associated with the higher frequency of gene mutation of BRAF, whereas the low *SPRED3* group of THCA patients had a higher NRAS mutations. Indeed, the BRAF (V600E) mutant in combination with its splicing variations may speed up the development of poorly differentiated THCA^27^. Recent genomic landscape analysis of TCHA have also shown that BRAF mutations occur in around 60% of PTC and 45% of ATC (Anaplastic thyroid carcinoma) regarding as driving mutations^28^. Additionally, NRAS mutations were common in advanced papillary thyroid cancer (PTC) and follicular thyroid cancer (FTC)^29^. The correlation of *SPRED3* expression with BRS, TDS and ERK further supported the oncogenic role in THCA. The results suggest that overexpression of *SPRED3* is associated with dyscohesive cells at the periphery of the tumor and at its invasive front, which contribute to tumor progression by activating the MAPK signaling pathway^30^. More importantly, *SPRED3* overexpression promoted THCA cell proliferation, while SPRED3 curbed the tumor cell proliferation and tumor growth in vivo. Therefore, we for the first time reveal the oncogenic characteristics of SPRED3 during THCA malignancy.

GESA analysis revealed a noticeable reduction in the enrichment of the NF-κB pathway upon the downregulation of *SPRED3* expression. To confirm these findings, we conducted a dual-luciferase reporter gene assay, which further supported the observed dampening effect of SPRED3 on the NF-κB pathway. NF-κB serves as a critical driver of malignant behaviors in cancer cells^19,31^. Our data demonstrated that SPRED3 facilitated the transcriptional activity of NF-κB. The NF-κB signaling cascade is tightly regulated by IkB^32,33^. Upon activation, NF-κB accumulates and translocates to the nucleus, thereby upregulating the expression of inflammatory effectors such as *TNFa, B94*, and *ICAM1*^34,35^. *B94*, named TNF alpha-induced protein 2 (TNFAIP2), is induced by the TNFa^36^. In our study, we observed a substantial increase in the expression levels of *TNFα, B94, and ICAM1* upon SPRED3 overexpression, and these effects were counteracted by TPCA-1, an inhibitor of the NF-κB signaling pathway^37^. NF-κB influences multiple physiological and pathological processes and plays a crucial role in the signal transduction of various cell-extrinsic cues^38^. Importantly, in cancer cells, aberrant NF-κB activation exerts control over inflammatory tumors, sustaining macromolecular production and cell survival even in the absence of growth stimuli, thus promoting the malignant characteristics of tumor cells^39^. Therefore, SPRED3 might cause NF-κB activation to increase tumor cell malignant phenotypes and thereby lead to tumorigenesis in THCA.

In short, we found that SPRED3 was overexpressed in THCA and confirmed its tumor-promoting role in partly activating the NF-κB signaling pathway. Our findings highlight the potentials of SPRED3 as a future molecular target against THCA.

## Methods

### TCGA cohort pan-cancer data collection

Using the R package TCGA biolinks, *SPRED3* mRNA expression profile data for TCGA pan-cancer were retrieved to analyze the differential expression levels of SPRED3 in cancer tissues and paired/ unpaired tumor-free tissues. Meanwhile, the clinicopathological information including patient outcomes was collected in the TCGA-THCA cohort to verify the clinical significance of SPRED3. *SPRED3* expression in THCA was further evaluated by the GSE33630 database.

### Assessment of SPRED3’s significance in the TCGA THCA cohort

Using the R package ‘survival’, the survival of the TCGA pan-cancer cohort was plotted to analyze the difference between the high-*SPRED3* and low-*SPRED3* groups. Logistic regression was conducted using the R package ‘survey’. The clinicopathological parameter data of patients in the THCA cohorts were obtained from TGCA, and a χ2 test using *R* language was used to analyze the association of SPRED3 with the clinicopathological parameters. Whether the *SPRED3* expression levels were associated with the traditional molecular markers of papillary thyroid cancer such as BRAFV600E-RAS score (BRS), Thyroid differentiation score (TDS) and ERK activation level (i.e., ERK score) was estimated in papillary THCA in these silico analysis.

### Gene enrichment analysis (GESA)

GESA analyses were conducted to enrich differentially expressed genes using the R package: fgsea and cluster Profiler.

### Gene mutation gene analysis

R ‘maftools’ package was utilized to analyze and visualize data on gene mutations. Oncoplots show and label the ten most frequently altered genes. In patients with low and high expression of SPRED3, the top seven statistically significant genes were compared.

### Immune infiltration analysis

Based on the TCGA RNA-seq dataset, the ESTIMATE algorithm and CIBERSORT were adopted to analyze the association of SPRED3 expression with the immune microenvironment (stromal score, immune score, ESTIMATE score, and tumor purity) and the immune cell infiltration. Estimate CIBETORT and TIMER ssGSEA were also used.

### Cells lines and cell culture

Two thyroid carcinoma cell lines (TPC-1 and BC-PAP) were commercially obtained from the cell back of the China Center for Type Culture Collection (CCTCC at Wuhan University) and maintained in low-glucose Dulbecco’s modified eagle medium (DMEM) (Pyruvate, Germany) at 37 °C with 5% CO_2_. The medium was supplemented with containing 10% fetal bovine serum (FBS) (Reboiosci, China) and 1% penicillin–streptomycin (Reboiosci, China).

### Establishment of SPRED3-overexpressing THCA cells

The human full-length SPRED3 cDNA was amplified from THCA cells using RT-PCR and ligated into the pHAGE puro 6tag constructs (Invitrogen, USA). The produced lentiviral constructs and the empty controls were packaged in 293T cells accompanied by packaging plasmids. Afterward, the medium containing lentiviral constructs was ultracentrifuged through 0.22 μm filtration. THCA cells were subjected to puromycin selection for 14 days following infection at a 1 multiplicity of infection (MOI). Finally, the survival clones were verified by western blots.

### Construction of SPRED3-deficient THCA cells

SgRNA inserts (sgRNA1 and sgRNA2) targeting SPRED3 Exon1 and Exon 2 (http://crispor.tefor.net/) were subcloned into lentiCRISPRv2 vectors (Addgene, USA). The produced CRISPR/Cas9 sgRNA viral vectors and the empty vectors (2 μg/mL) were transfected with 75% confluent 293T cells. The amplified vrial particles infected THCA cells and were selected under puromycin (5 µg/mL). The knockout-1 (KO-1) and knockout-2 (KO-2) were selected and confirmed by western blot. The SPRED3 knockout was verified by Western blot. sgRNA-1 sequence: TTCCGGCGCGCCGAGTCCTT AGG sgRNA-2 sequence: CGCCGGCGCGCGCCCAGATT GGG. The uninfected THCA cells served as the control cells.

### Measurement of cell proliferation

THCA cells (1 × 10^4^ cells/well) were plated on 96-well plates. After 24 h, 48, and 72 h maintenance, THCA cells were exposed to 10 μL CCK8 reagent (Beyotime, China). Another 3 h later, the plates were read by a microplate spectrophotometer at 450 nm.

Furthermore, the THCA cell proliferation was also assessed by colony formation assays. 500 THCA cells were inoculated in 60-mm plates. 10 days later, THCA cells were stained with 0.1% crystal violet (Sigma) for 30 min after being fixed with paraformaldehyde fixative for 30 min. Viable colonies of more than 50 cells were calculated.

### Luciferase reporter assays

NF-κB luciferase reporter plasmid (Jubio, Shanghai, China) was used to determine whether SPRED3 interacted with NF-κB as predicted in GESA analysis. As instructed, the NF-κB luciferase reporter plasmid was delivered into the SPRED3-overexpressing 293T cells using Lipofectamine 2000 (Invitrogen). After 48 h, the luciferase activity was measured using the Promega luciferase system. Furthermore, the NF-κB luciferase reporter plasmid was transfected into the SPRED3-deficient TPC-1 cells to verify the change of NF-κB transcriptional activity when SPRED3 deficiency.

### RT-PCR analysis

The total RNA was isolated from THCA cells Trizol (TRIzol, Invitrogen) and subjected to reverse transcription using Qiagen QuantiTect Reverse Transcription Kit (Qiagen, China). The quantification of the target genes was done using Roche LightCycler 480 system with SYBR Green reagents (Takara, Japan). With normalization to GAPDH, the outcomes were analyzed by the 2^−ΔΔCt^ method. The primers were listed as below: *B94*: Forward primer 5′-CTGCATCTGCGAGAAGAGGG-3′, Reverse primer 5′-ACATGGAGGCCTCTGACTCT-3′; *TNFa*: Forward primer 5′-GCATGCCAGTCAGGTAGTGT-3′, Reverse primer 5′-TCGGTGAGCAGTTTGTCTCC-3′. *ICAM1*: Forward primer 5′-GAAATCATGTCTTGTGGAACTGA-3′, 5′-CTCCTTCAACAGAGAAGCCAG-3′; IκBa: Forward primer 5′-TGTGCTTCGAGTGACTGACC-3′, Reverse primer 5′-TCACCCCACATCACTGAACG-3′. SPRED3: Forward primer 5′-TGGACTGACGTTTCAGAGC-3′, Reverse primer 5′-CCTGAAGCTGACTCCATCGT-3′ GAPDH: Forward primer 5′-CTCAGACACCATGGGGAAGGTGA-3′, Reverse primer 5′-ATGATCTTGAGGCTGTTGTCATA-3′.

### Western blot

THCA cells were treated with the prechilled lysis buffer (Byotime, China). The harvested supernatant was quantified by a BCA kit (Pierce, USA). 10 μg proteins were separated by 10% SDS-PAGE and then loaded onto PVDF membranes which are blocked in 5% fat-free milk powder. At 4 °C, the primary antibodies were used for SDS-PAGE to blot target proteins. The HRP-linked secondary antibody (ZSGB-BIO, China) was utilized for the valuation of proteins at room temperature for 1 h. The antibodies used were listed as below: anti-NFκB (Cat#3034, 1:1000, Cell Signaling Technologies; Danvers, MA), Anti-GAPDH antibody (Cat#. ab9484; 1:1000; Abcam); anti-p-p65 (Cat#3033, 1:1000, Abcam), anti-p-p65 antibody (Cat#F3165; 1:1000, Sigma-Aldrich, Merck), anti-SPRED3 antibody (Cat#bs-20643R, 1:1000, BioSS, China), Goat Anti-Rabbit IgG H&L/HRP (Cat#bs-0295G-HRP, 1:1000, BioSS, China)(Supplementary Figures).

### Mouse xenograft studies

Eighteen Balb/c nude mice at ~ 6–7 weeks of age were purchased from the Experimental Animal Center of Medicine College of Wuhan University (Wuhan, China). The mice were grouped into three groups (n = 6): KO1, KO2, and WT. Two KO1 and KO2 groups were injected subcutaneously with 0.5 × 10^6^ each of SPRED3-deficient TPC-1 cell clones on Day 0. The mice in the WT group were injected with wild-type TPC-1 cells. Every week, Tumor size was monitored using Vernier caliper. Following the mice receiving the CO_2_ asphyxiation per protocol, the tumor was dislocated and weighted. Animal ethics approval was granted by the ethical committee of Fujian Medical University. All animal experiments were reported according to the ARRIVE guidelines. All methods were performed in accordance with the relevant guidelines and regulations.

### Statistics

Results were expressed as mean ± SD. Prism 9.0 was adopted for outcome analysis. Paired or unpaired student *t*-test was undertaken to compare the differences between the two groups. The difference among multiple groups was analyzed using One-way ANOVA. A value of *p* < 0.05 was considered a statistical significance. Experiments were repeated three times with three replicates.

### Ethics approval and consent to participate

Animal ethics approval was granted by the ethical committee of Fujian Medical University. All animal experiments were reported according to the ARRIVE guidelines. All methods were performed in accordance with the relevant guidelines and regulations.

## Supplementary Information

Supplementary Figures.

## Data Availability

All data generated or analyzed during this study are included in this published article.
