# Supplementary figures and images for: SPRED3 regulates the NF-κB signaling pathway in thyroid cancer and promotes the proliferation
Source: Sci Rep. 2024 Sep 3;14:20506. doi: 10.1038/s41598-024-61075-6 (PMC11372091; doi:10.1038/s41598-024-61075-6)

## Slide 1
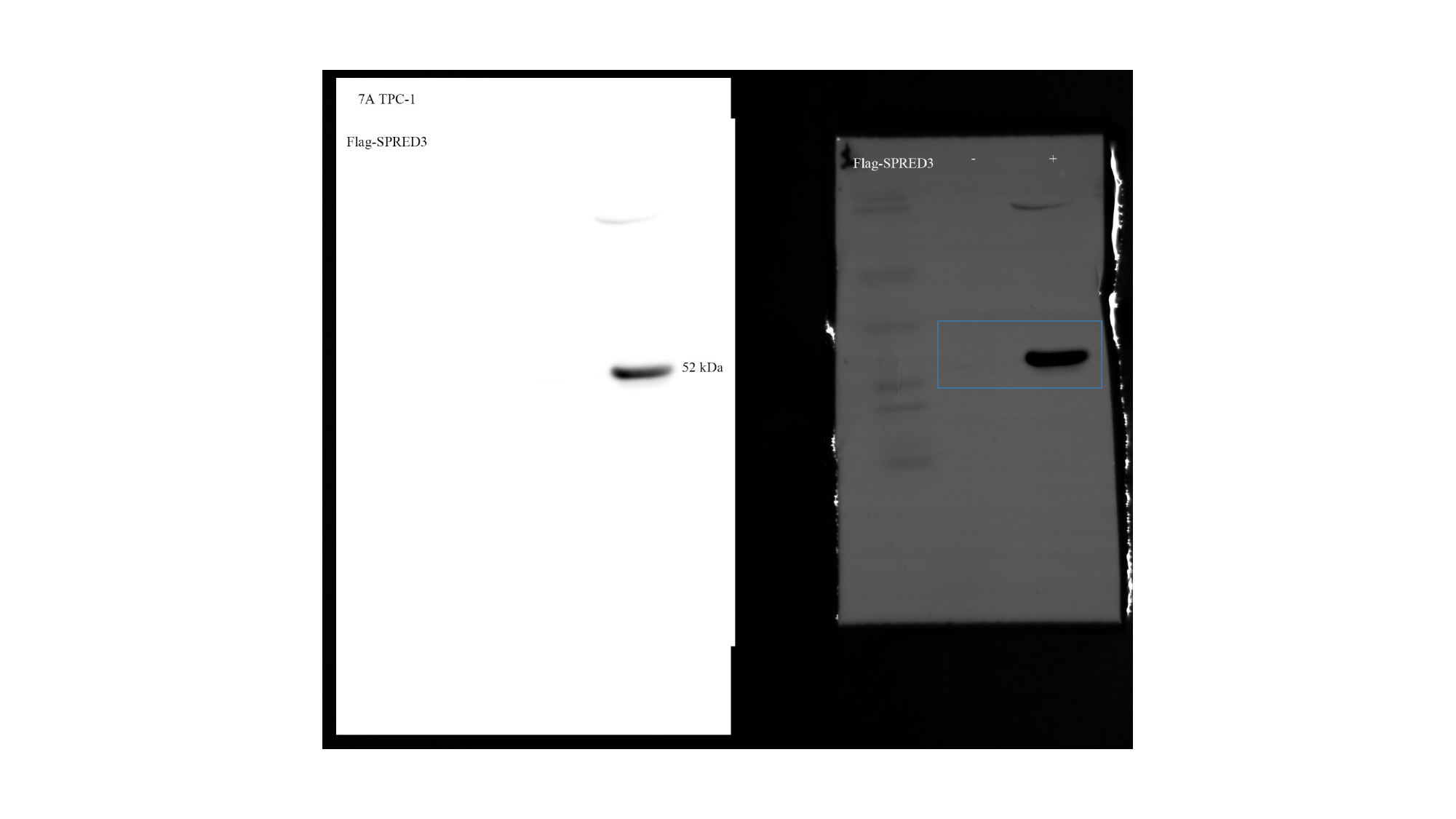

## Slide 2
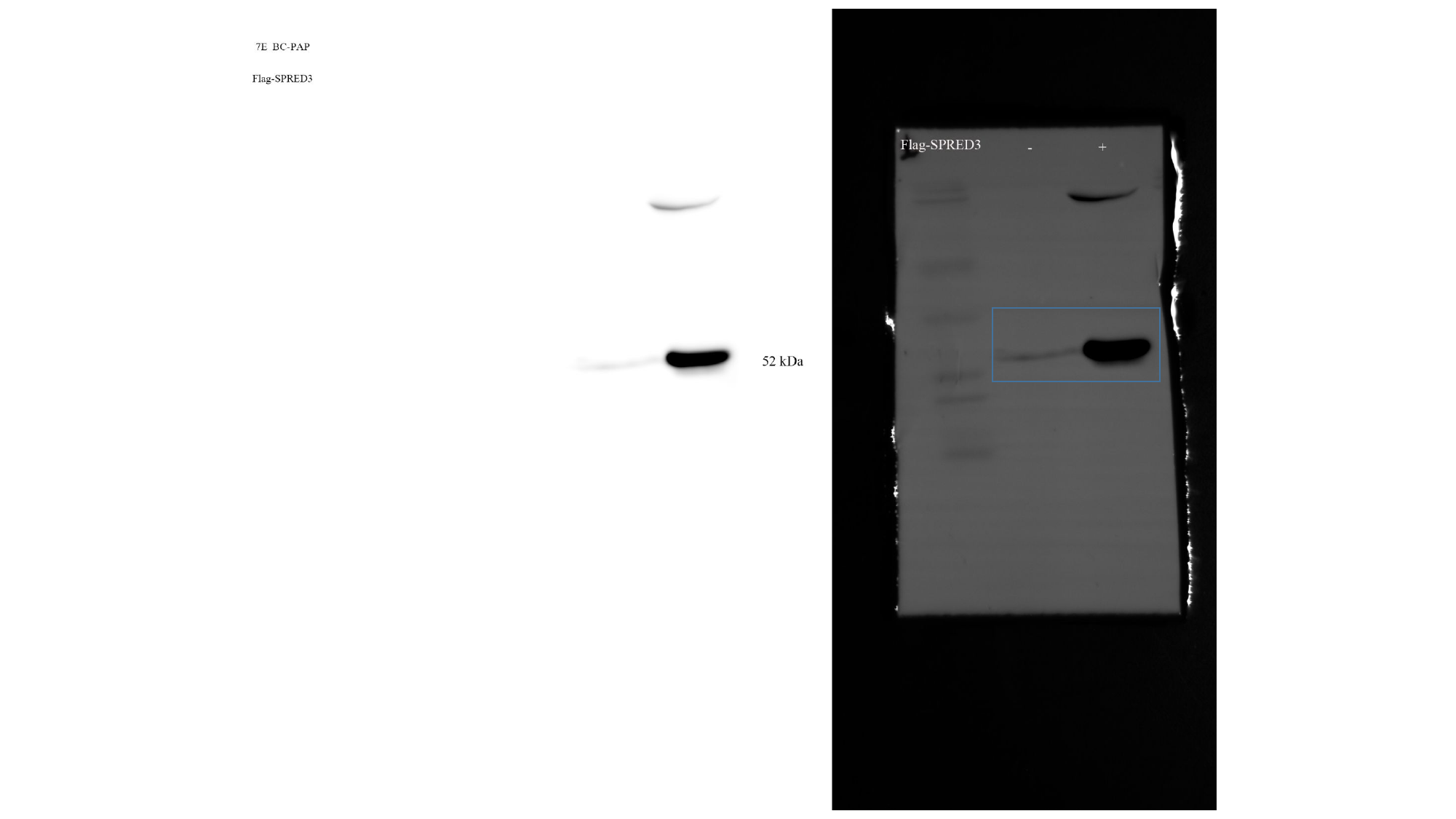

## Slide 3
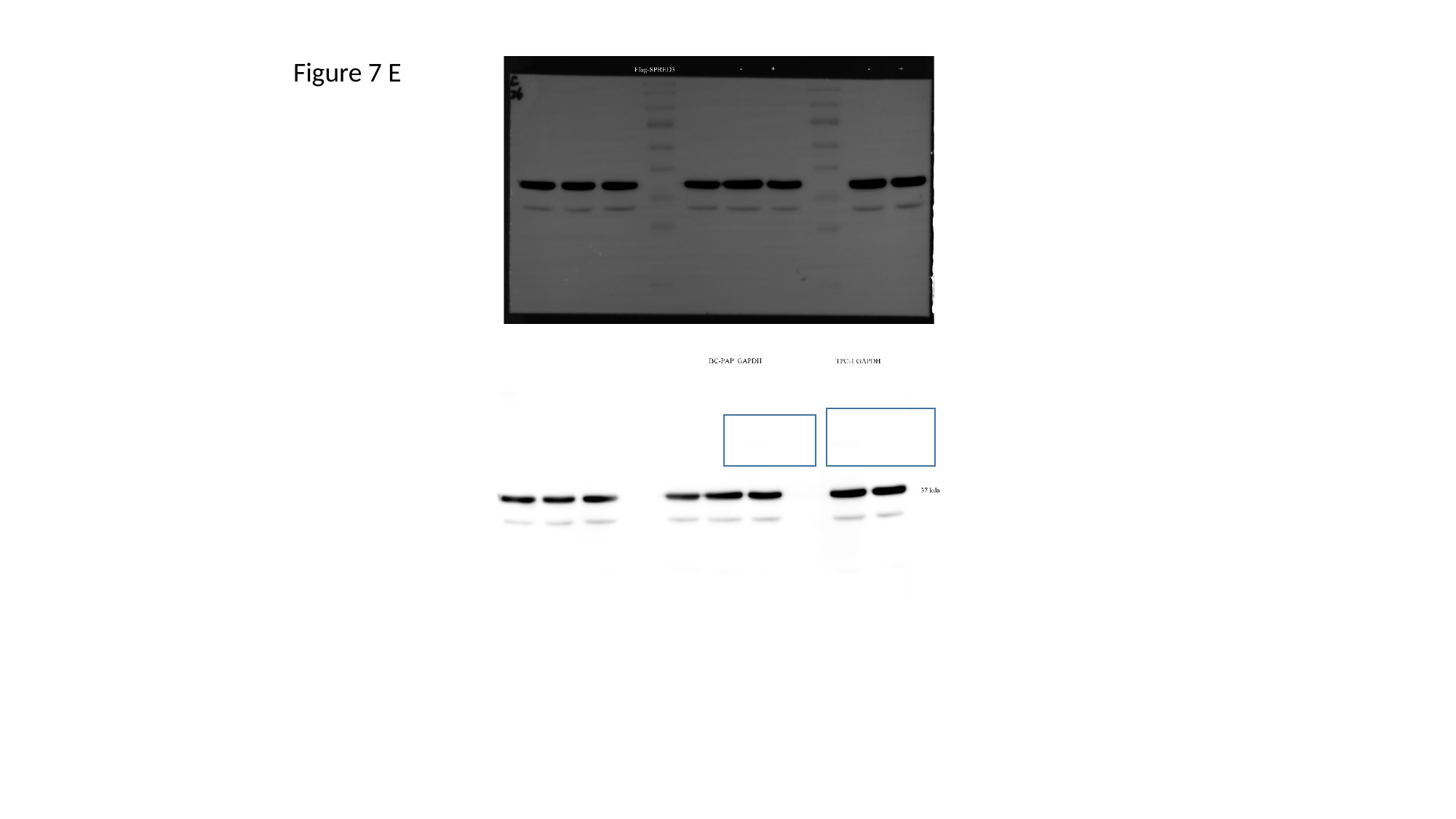

Figure 7 E

## Slide 4
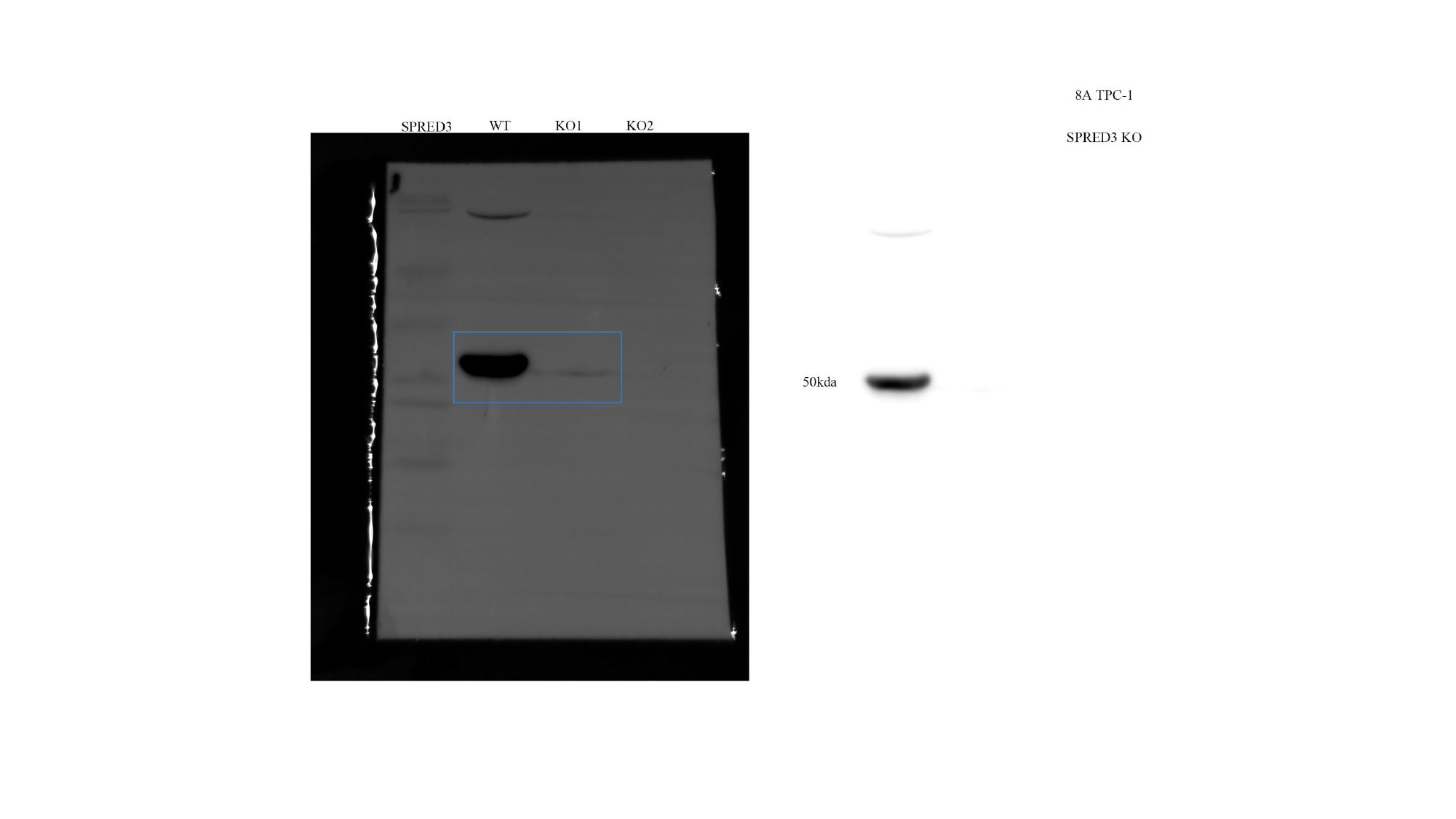

## Slide 5
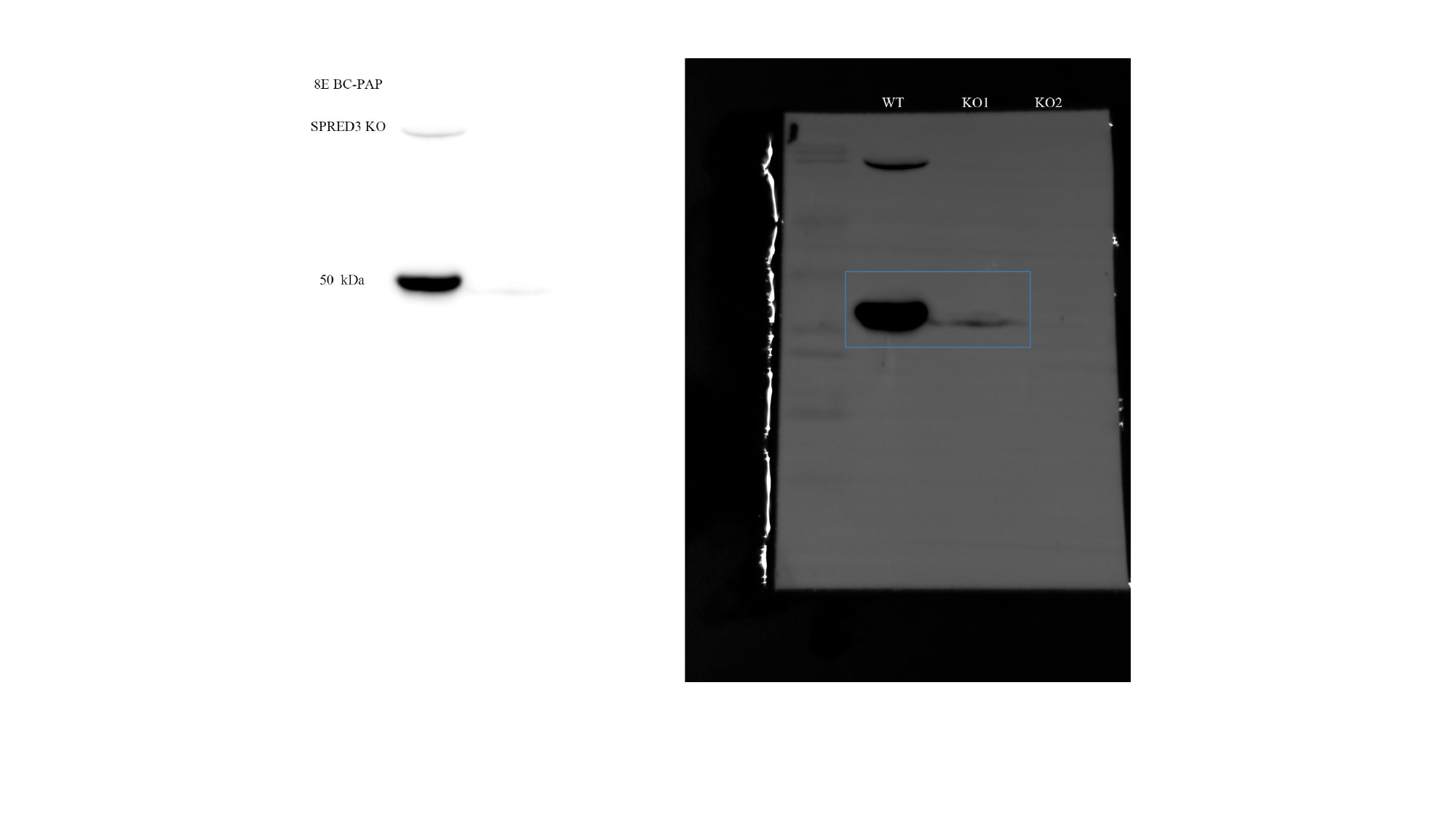

## Slide 6
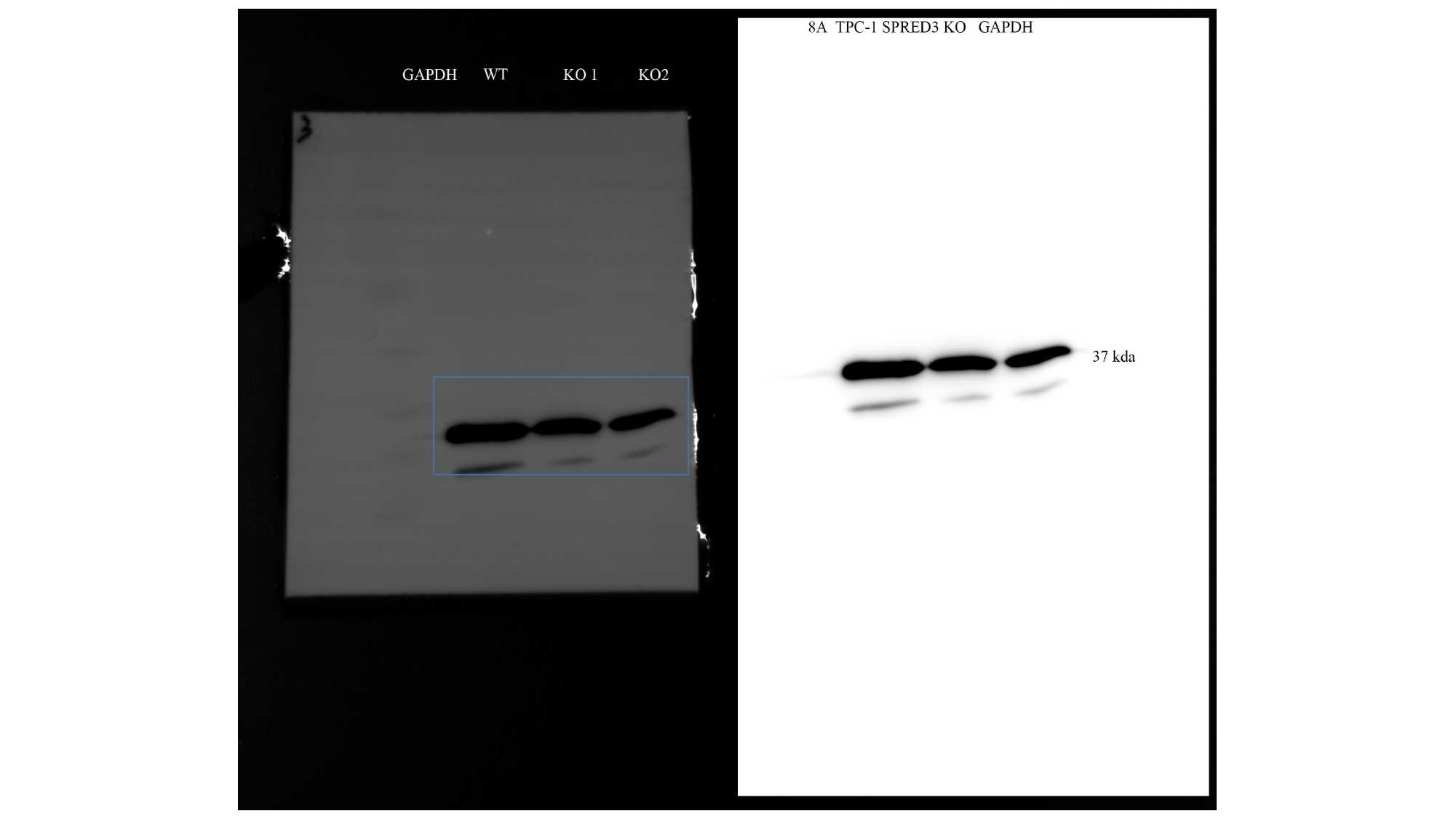

## Slide 7
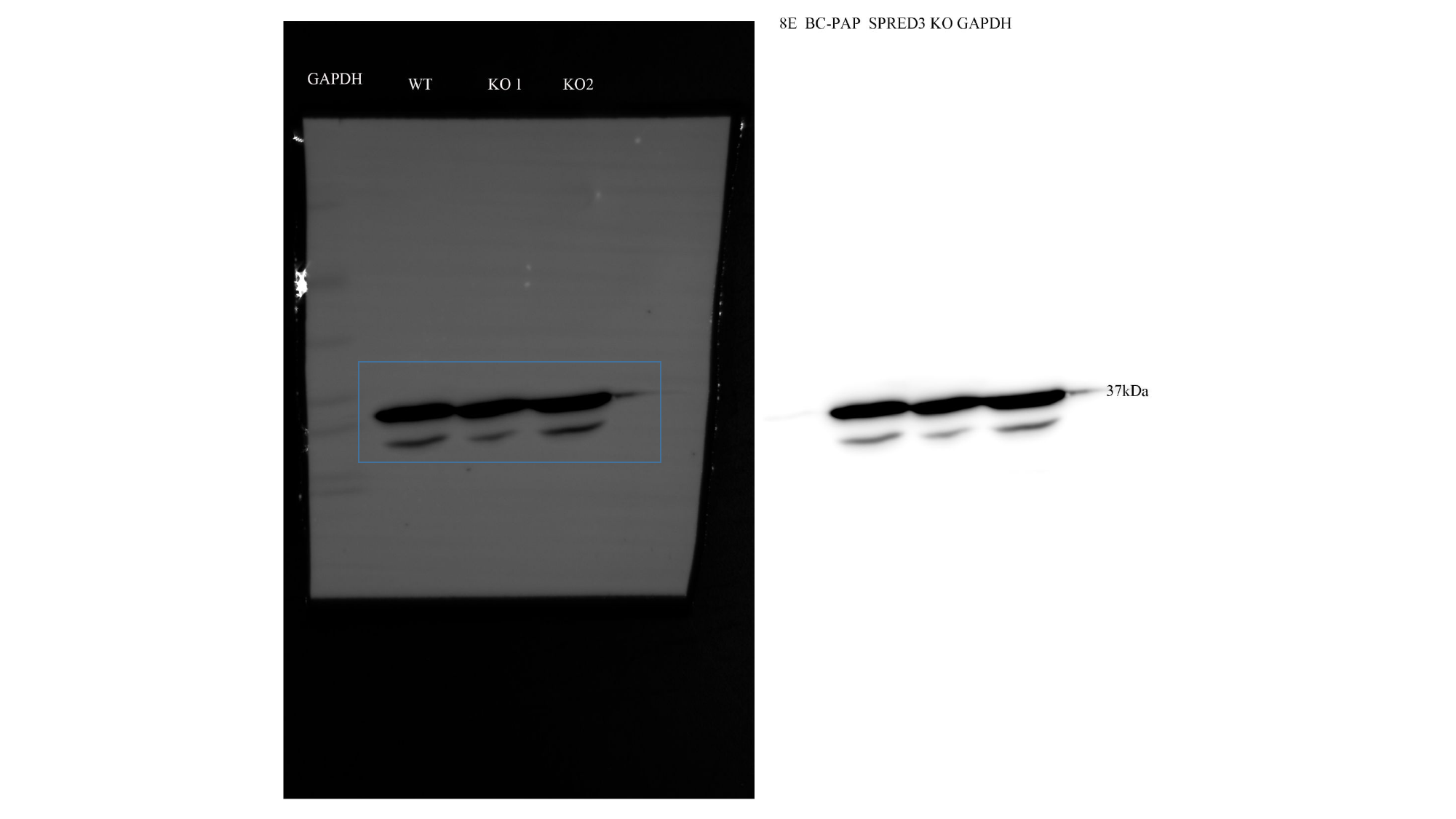

## Slide 8
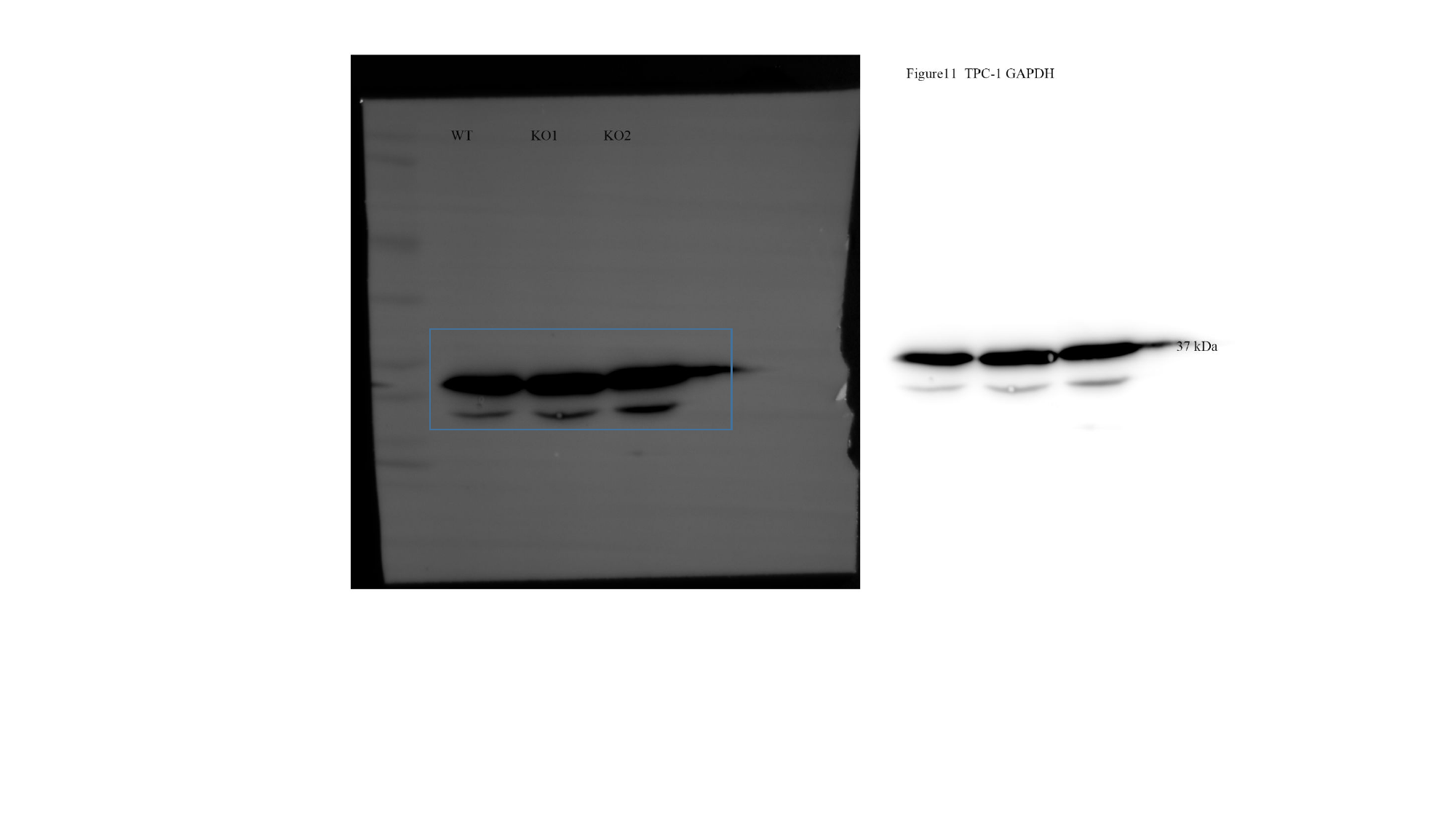

## Slide 9
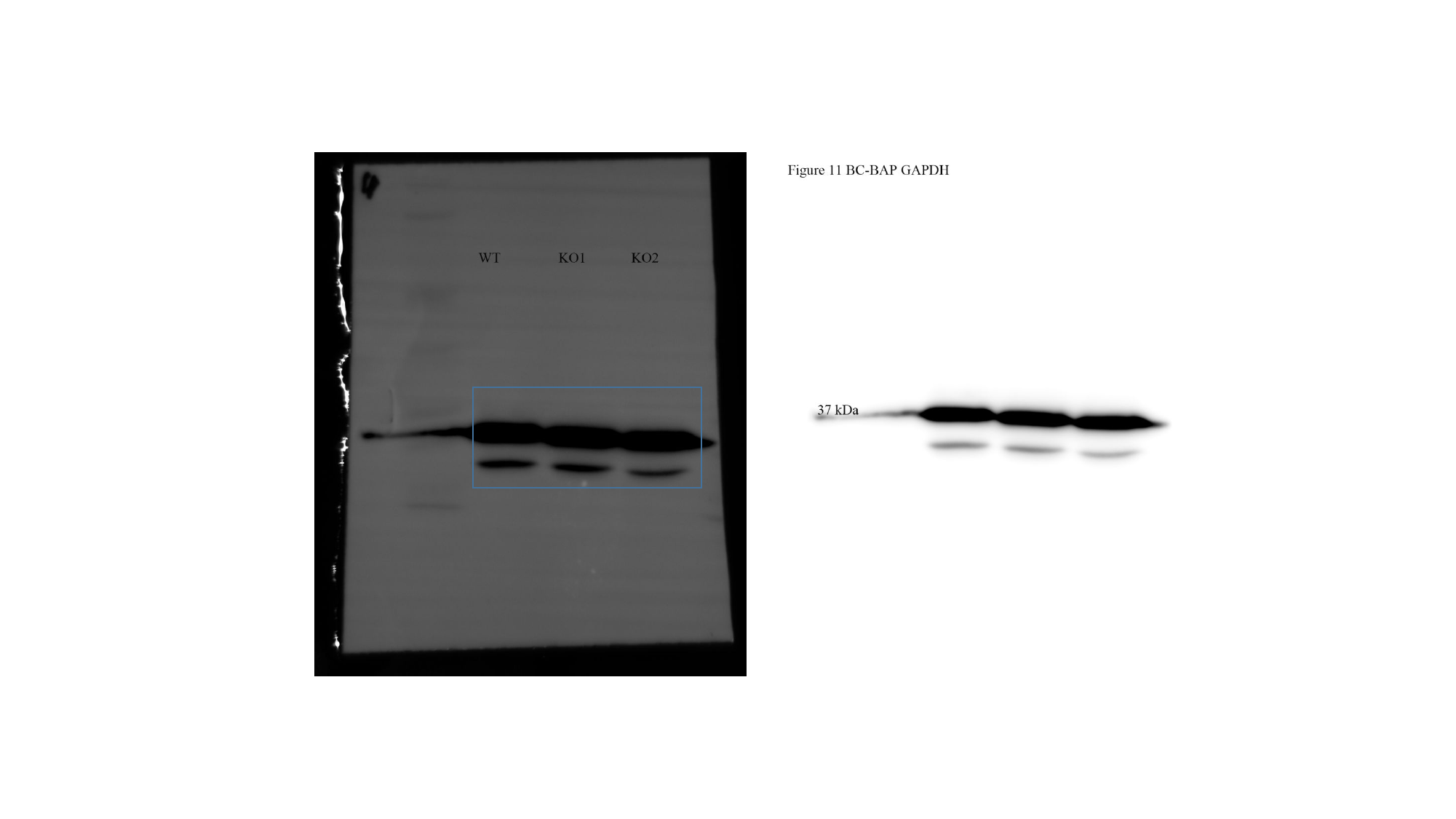

## Slide 10
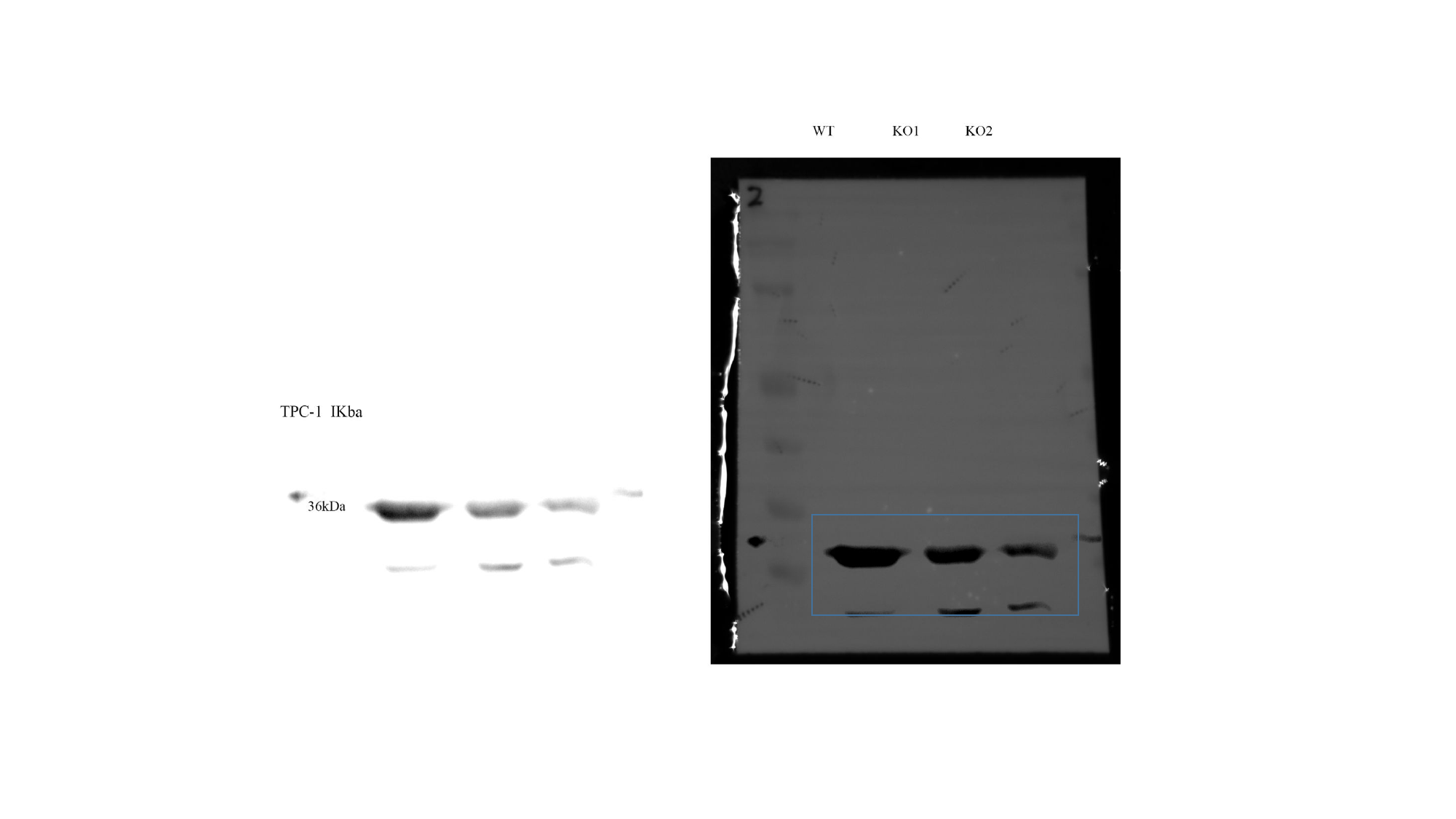

## Slide 11
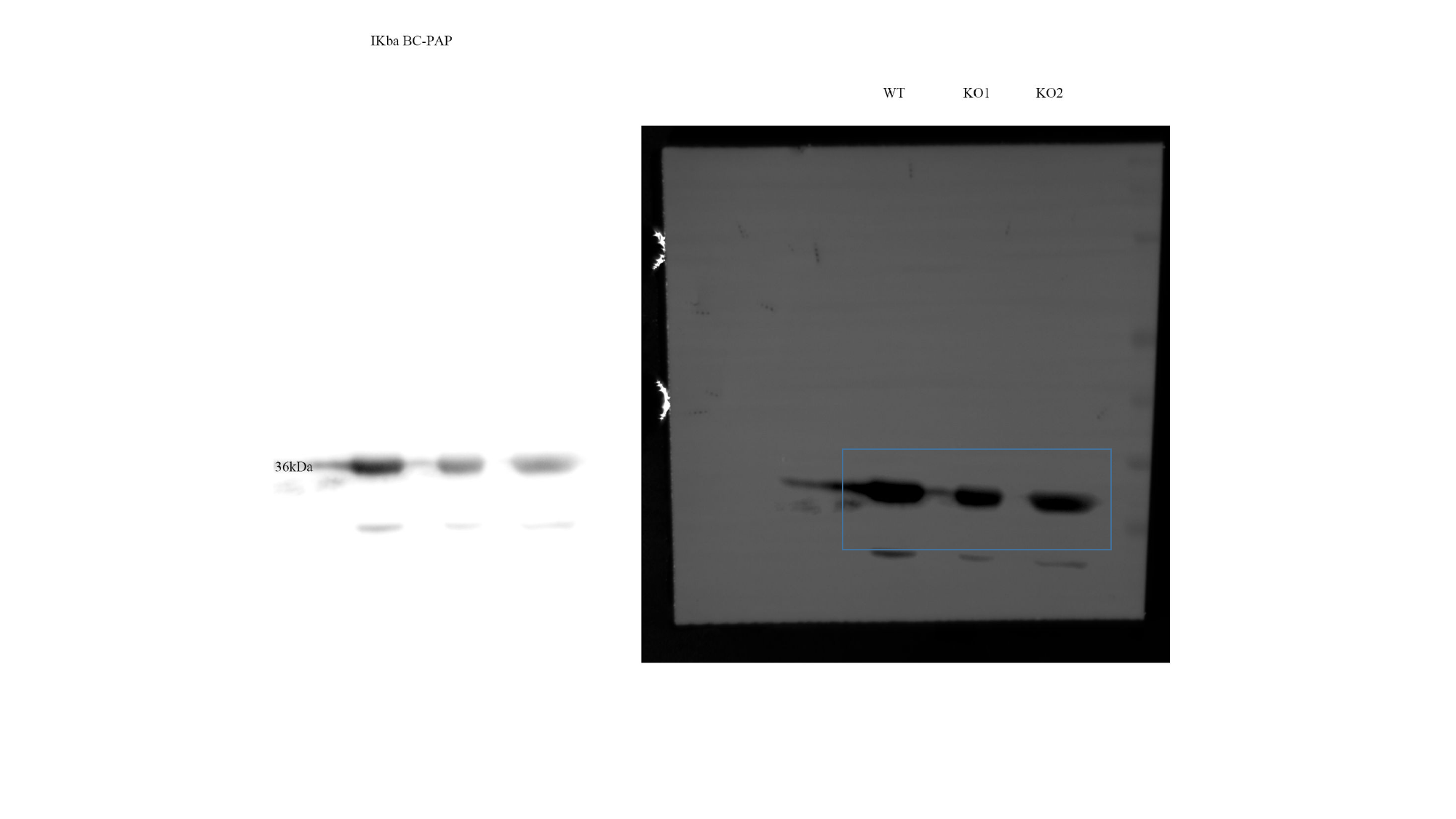

## Slide 12
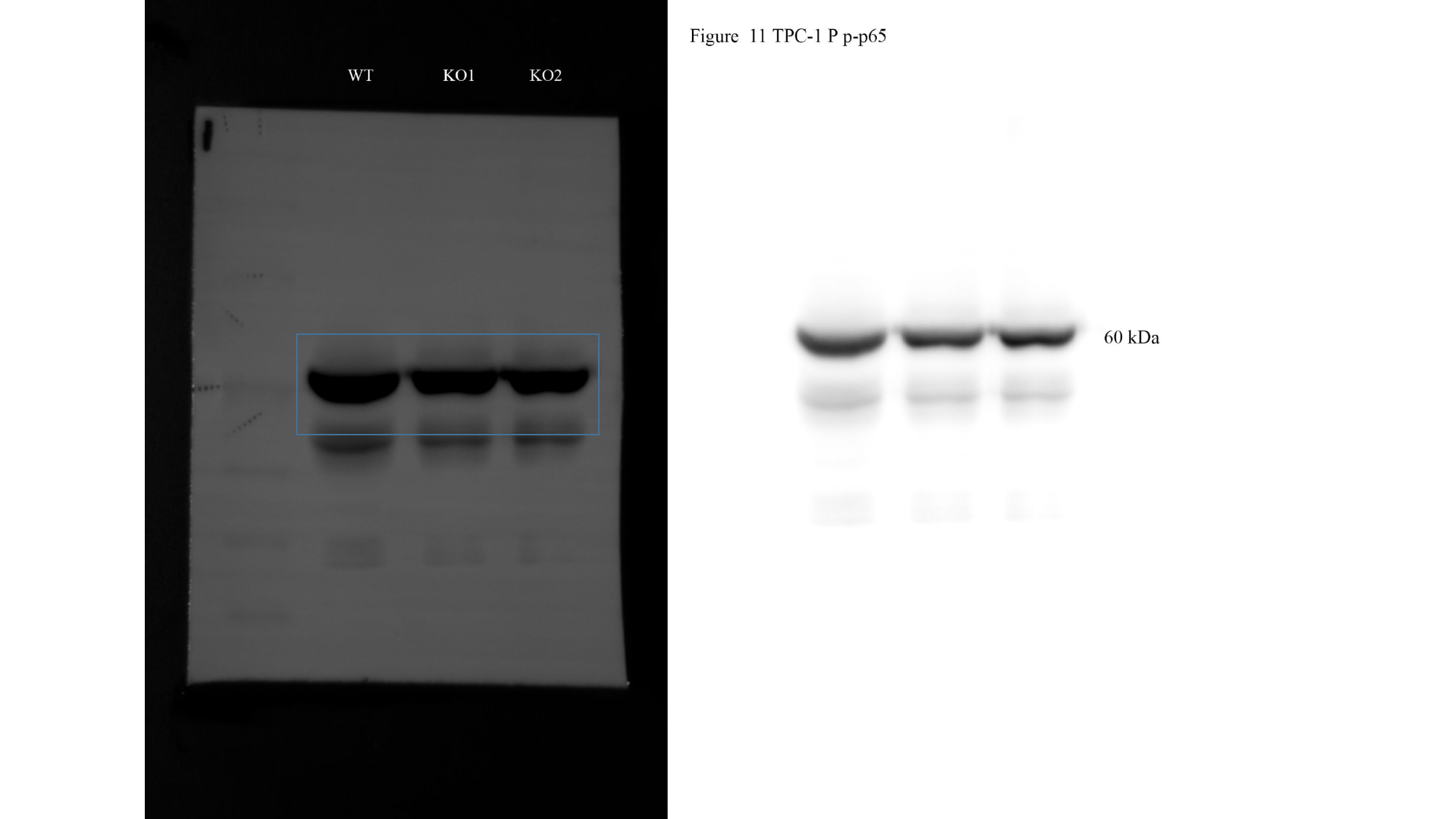

## Slide 13
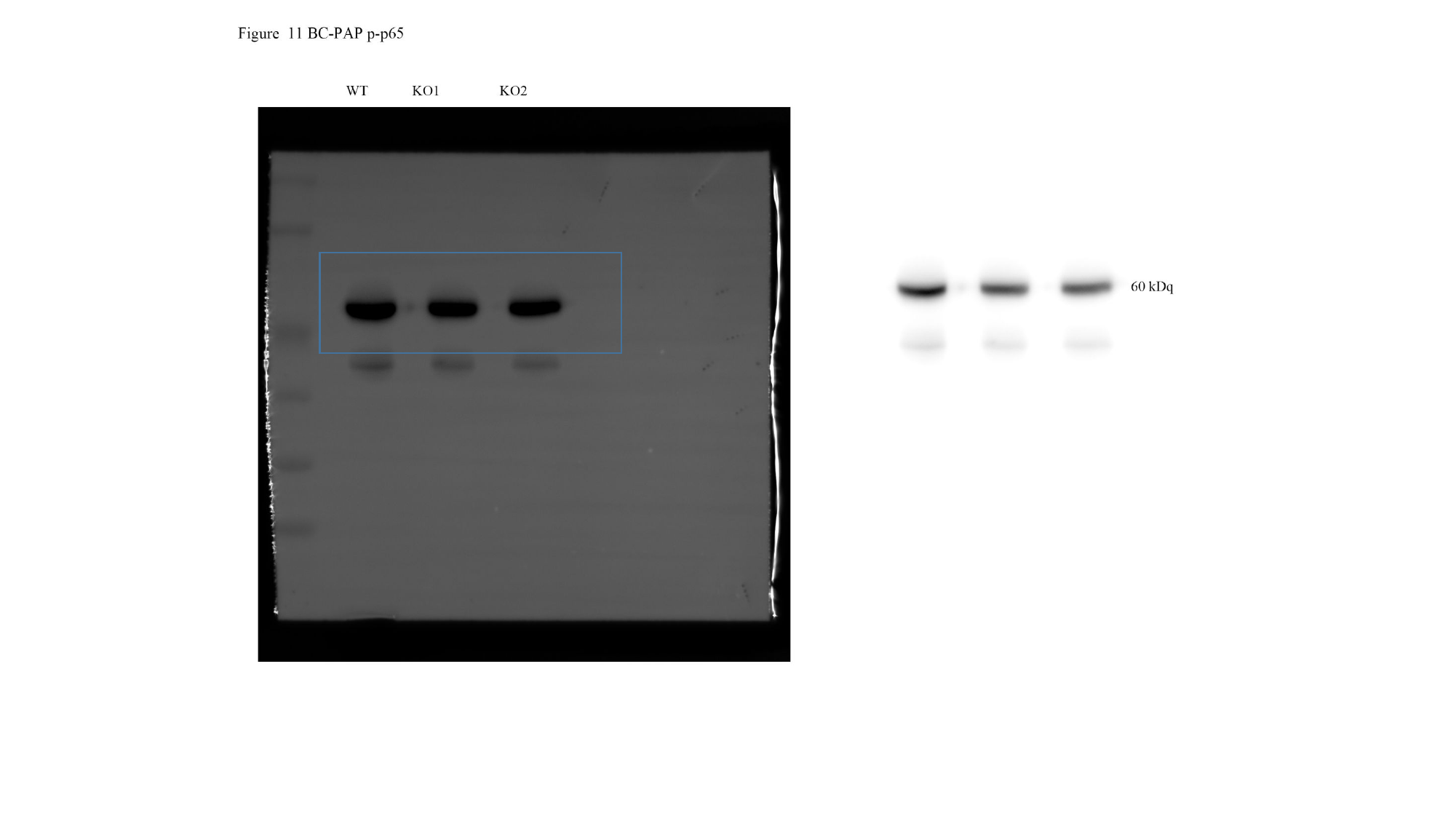

Supplement: Supplementary file 1 — Supplementary Figures. [file 41598_2024_61075_MOESM1_ESM.pptx]
